# Space-time clustering and temporal trend analysis of pulmonary tuberculosis in Brazil, 2010–2023

**DOI:** 10.1371/journal.pone.0339784

**Published:** 2026-01-08

**Authors:** José Mário Nunes da Silva, Fredi Alexander Diaz-Quijano, Lívia Teixeira de Souza Maia, Mauro Niskier Sanchez, Ximena Pamela Claudia Díaz Bermúdez, Eduardo de Souza Alves, Walter Massa Ramalho

**Affiliations:** 1 Center for Tropical Medicine, School of Medicine, University of Brasília, Brasília, Federal District, Brazil; 2 Department of Epidemiology – Laboratório de Inferência Causal em Epidemiologia (LINCE-USP), School of Public Health, University of São Paulo, São Paulo, São Paulo, Brazil; 3 Center for Public Health, Federal University of Pernambuco, Vitória de Santo Antão, Pernambuco, Brazil; 4 Department of Public Health, School of Health Sciences, University of Brasília, Brasília, Federal District, Brazil; 5 Ministry of Health, Brasília, Federal District, Brazil; Saint Mary's University, CANADA

## Abstract

**Background:**

Identifying high-risk areas for pulmonary tuberculosis (PTB) is essential for understanding the dynamics of disease transmission and for guiding more effective control strategies. Therefore, this study aimed to analyze the temporal trends and spatiotemporal distribution of PTB across Brazilian municipalities between 2010 and 2023.

**Methods:**

This is an ecological study using data on PTB cases reported in Brazil’s Notifiable Diseases Information System (SINAN) from 2010 to 2023. Time series analysis, global and local spatial autocorrelation, and space-time scan techniques were applied to identify temporal trends and spatial patterns of the disease at the municipal level.

**Results:**

A total of 896,062 new PTB cases were analyzed. Notification peaked mainly in March and August. The average incidence rate was 30.3 cases per 100,000 inhabitants-years. An increasing trend was observed in 13 states and the Federal District. Spatial analysis identified 804 municipalities as hotspots, with 168 showing persistent high incidence throughout the study period. A total of 28 spatiotemporal clusters were detected, involving 379 municipalities, of which 212 were classified as high priority. The most likely cluster was located in the Rio de Janeiro Metropolitan Region, encompassing 10 municipalities, with a relative risk (RR) of 2.51 between 2017 and 2023. Spatial variation in temporal trends identified 22 additional clusters, including a prominent cluster composed of 493 municipalities in the Legal Amazon, which showed an internal time trend of 1.99% annual growth and a RR of 1.51.

**Conclusion:**

The study identified persistent and expanding patterns of PTB in Brazil at both regional and national levels, revealing specific areas with higher burden and increasing trends that should be prioritized. These findings provide evidence to support decision-making at federal level while reinforce the need for regionally tailored surveillance and control strategies to ensure a more effective and equitable response to tuberculosis across the country.

## Introduction

Tuberculosis (TB) has once again become the leading cause of death from a single infectious agent worldwide, accounting for an estimated 1.25 million deaths in 2023 [1]. In the same year, Brazil reported 84,994 new TB cases, and TB-related mortality, after declining up to 2020, resumed an upward trajectory, reaching 6,025 deaths [2]. Despite the progress achieved in recent decades, global efforts remain insufficient to meet the goal of eliminating TB as a public health problem by 2035 [3].

As an airborne infectious disease, pulmonary tuberculosis (PTB) has a high potential for dissemination, affecting not only areas with high incidence but also adjacent regions [4]. In a country as large and diverse as Brazil, TB distribution is highly heterogeneous, reflecting deep social and structural inequalities. The persistence of poverty, housing shortages, overcrowded environments, malnutrition, and limited access to health services contributes to maintaining endemic transmission, particularly in urban peripheries and territories with poor living conditions [5–7]. Incarceration, migration, and informal labor further exacerbate vulnerability, while regional disparities in health-system capacity and social protection reinforce unequal exposure and outcomes [8,9].

These inequalities explain why certain municipalities and regions, such as large metropolitan areas, border zones, and parts of the Legal Amazon, have remained persistent TB hotspots over time [5,6,10,11]. Identifying these spatial disparities is therefore crucial for advancing toward equitable TB control and aligning public health strategies with the broader social determinants of health.

In this context, spatial and spatiotemporal cluster analyses are well-suited to this task because they detect areas with more cases than expected and reveal patterns of persistence and spread [6,12]. Moreover, examining regional variations in disease trends allows for the evaluation of current interventions and helps identify where prevention and control strategies have yet to achieve the desired impact [5,7]. From a health management perspective, this approach supports the updating and adaptation of public health policies and enhances the allocation of resources to areas of highest priority [4,7,13]. Thus, this study aimed to analyze the temporal trends and spatiotemporal distribution of PTB in Brazilian municipalities between 2010 and 2023.

## Methods

### Study design and setting

This is an ecological study with mixed design, using Brazil’s 5,570 municipalities as units of analysis. Brazil, located in South America, is divided into five major geographic regions (North, Northeast, Central-West, Southeast, and South), which encompass 26 states and the Federal District [14]. Within this national territory, there is an area known as the Legal Amazon, which comprises 772 municipalities distributed across all the states in the Northern Region (Amazonas, Acre, Rondônia, Roraima, Amapá, Tocantins, and Pará), as well as the state of Mato Grosso (Central-West) and part of Maranhão (Northeast). This areas accounts for approximately 58.9% of the national territory and is home to 77% of the country’s Indigenous population (S1 Fig in S1 File) [15].

### Study population and data source

The study encompassed all newly reported cases of PTB between 2010 and 2023 in the Brazil’s Notifiable Diseases Information System (SINAN), managed by the Department of Informatics of the Unified Health System (DATASUS) (http://www2.datasus.gov.br). SINAN is the Brazilian system responsible for recording and processing data related to notifiable diseases nationwide, such as TB, and plays a key role in public health surveillance [16].

Census and intercensal population data for 2010–2023 were obtained from DATASUS, sourced from the Brazilian Institute of Geography and Statistics (IBGE) (https://www.gov.br/saude/pt-br/composicao/seidigi/demas/dados-populacionais).

### Variables

The incidence rate of PTB was calculated as the total number of reported cases from 2010 to 2023 divided by the resident population over the same period, expressed per 100,000 inhabitants per year. Records with missing sex (n = 76; 0.008%), age (n = 235; 0.03%), or municipality of residence (n = 168; 0.02%) were handled by proportional redistribution: (i) for sex and age, within the same municipality and calendar year; and (ii) for municipality of residence, within the same state and calendar year. This is equivalent to single imputation using empirical frequencies under missing completely at random (MCAR) or missing at random (MAR) assumptions, preserving totals and avoiding artificial rate deflation [17]. Two caveats apply: it does not propagate imputation uncertainty and could be biased if missingness were missing not at random (MNAR) [18]. However, given the extremely low missingness, uniform patterns across strata, and the data-collection workflow, we find no evidence of an MNAR mechanism [19].

The epidemiological characteristics of the cases included: sex, age (in years), self-reported race/ethnicity, education in years, area of residence, smoking status, alcohol consumption, illicit drug use, presence of comorbidities such as AIDS, diabetes mellitus, and mental disorders, as well as HIV and bacteriological status.

### Statistical analysis

#### Time series analysis.

We initially constructed a monthly time series of PTB cases to analyze their behavior and trends over the study period. For this purpose, we applied the Seasonal Trend Decomposition using Loess (STL) method, which is based on locally weighted regression [20]. This analysis was conducted using R software (version 4.4.2, R Core Team, Vienna, Austria), with the support of the forecast package.

In addition, we used the Joinpoint Regression Program (version 5.3.0.0, National Cancer Institute, Bethesda, MD, USA) to assess temporal trends. This model identifies whether a given indicator exhibits a stationary, increasing, or decreasing trend and determines the time points at which changes in these trends (joinpoints) occur. The following parameters were adopted for this analysis: log-linear regression model, minimum of 0 joinpoints, maximum of 3 joinpoints, model selection based on the Monte Carlo permutation test with 4,499 permutations, and first-order autocorrelated errors based on the data [21]. These settings enabled the estimation of the Annual Percent Change (APC) and the Average Annual Percent Change (AAPC), both with 95% confidence intervals (95% CI) and a significance level of 5%.

#### Spatial autocorrelation analysis.

We used the Global Moran’s Index (Moran’s I) to identify spatial autocorrelation and detect the spatial distribution pattern of PTB across Brazilian municipalities [22]. Moran’s I values range from −1 to +1: a positive value indicates positive spatial correlation, while a negative value suggests negative correlation, with high and low values interspersed [23]. Values near zero indicate no spatial clustering, suggesting that the data are randomly distributed [23]. To assess the significance of the spatial autocorrelation, we calculated the z-score and p-value using randomization procedures with 999 iterations via Monte Carlo simulations.

Subsequently, we applied the Local Getis-Ord *G*_i_^*^ statistic to identify local spatial autocorrelation and determine the location of clusters or spatial concentration zones (hotspots and coldspots). This analysis considered a neighborhood matrix based on the average number of events in the six nearest neighboring municipalities [24]. The output of this analysis included a z-score and corresponding p-value for each municipality. High positive z-scores indicated clusters of high values (hotspots), while negative z-scores indicated areas of lower occurrence (coldspots) [24]. A z-score above ±1.96 was interpreted as statistically significant, indicating a *p*-value < 0.05. All analyses were also performed using R software, employing spatial autocorrelation packages such as spdep and sfdep.

#### Space-time scan statistic analysis.

To identify spatiotemporal clusters of municipalities at higher risk of PTB transmission, we used Kulldorff’s space-time scan statistic [25]. This approach is based on a discrete Poisson probability model and a maximum likelihood ratio test [26]. The method involves the use of a moving cylindrical window, where the base represents the geographic area and the height corresponds to the temporal dimension [27]. This process allows for the identification of clusters in specific areas and the assessment, during a given time period, of whether there is a higher or lower proportion of cases compared to other areas under analysis [28].

Additionally, we applied the Spatial Variation in Temporal Trends (SVTT) technique, which differs from the previous methods by evaluating temporal trends within the clusters. The temporal trend is analyzed both inside and outside the scanning circle: the variation in trend within the cluster is referred to as the internal time trend (ITT), while the trend in the surrounding areas is termed the outside time trend (OTT). In this analysis, statistical significance is assessed for the temporal trends (ITT and OTT), rather than for cluster formation as in spatial and space-time scanning [7].

In these analyses, the relative risk (RR) is calculated as the ratio between the estimated risk inside the cluster and the estimated risk outside the cluster [26]:


$$RR=\;\frac{c/E[c]}{\left(C-c)/(C-\;E[c]\right)}$$

Where *c* is the number of observed cases within the cluster, *C* is the total number of cases within the scanning window, and *E[c]* is the expected number of cases, adjusted for covariates, under the null hypothesis.

Similarly, the logarithmic likelihood ratio (LLR) is calculated for various circular window centers and radii by comparing PTB incidence rates inside and outside the window [26]:


$$LLR=\text{log}{\left(\frac{c}{E[c]}\right)}^{c}{\left(\frac{C-c}{C-\;E[c]}\right)}^{C-c}.\;I()$$

Where (*C* − *E[c]*) is the expected number of cases outside the window, and *I()* is an indicator function.

Thus, the window with the highest LLR value was identified as the most likely cluster, while secondary clusters were defined as other windows with statistically significant LLR values. Statistical significance was evaluated through Monte Carlo simulations (999 replications), and clusters with a *p*-value < 0.05 were considered statistically significant [26].

To mitigate statistical noise and enhance the interpretability of results, clusters composed of two or fewer municipalities were excluded from the analysis, as recommended [26]. Very small clusters are more susceptible to random variability and to the modifiable areal unit problem (MAUP), which can lead to unstable incidence estimates and potentially spurious patterns or false positives arising from random fluctuation [5,29]. In this analysis, the maximum radius of the cylinder base was set to encompass up to 50% of the total at-risk population, and the maximum temporal size of clusters was limited to 50% of the study period. Age group and sex of the reported cases were included as covariates. This step was performed using SaTScan software (version 10.2.5, National Cancer Institute, Bethesda, MD, USA).

All the cartographic bases used for constructing the thematic maps were obtained from the official and publicly available database of the IBGE (https://www.ibge.gov.br/geociencias/organizacao-do-territorio/malhas-territoriais.html). These shapefiles are open-access and released under public domain. All maps presented in this study were produced by the authors using these public datasets.

### Ethical statement

This study utilized only publicly available secondary data in aggregate form, with no potential for individual identification. Thus, the study did not require informed consent or review by an Ethics Committee.

## Results

### Descriptive analysis

Between 2010 and 2023, 1,042,692 new TB cases were reported in Brazil, of which 896,062 were PTB (S2 Fig in S1 File). Overall, 620,704 cases occurred in men (69.3%), yielding a male-to-female ratio of 2.25:1. The median age was 37 years (interquartile range [IQR], 26–52), and 221,758 cases were recorded in the 20–29-year age group (24.7%). In addition, 538,706 cases (60.2%) occurred among individuals who self-identified as mixed race (*pardo*) or Black, and 652,423 cases (72.8%) were bacteriologically confirmed. Additional demographic and clinical characteristics of PTB cases are presented in S1 Table in S1 File.

### Temporal patterns and trend

**Fig 1:**
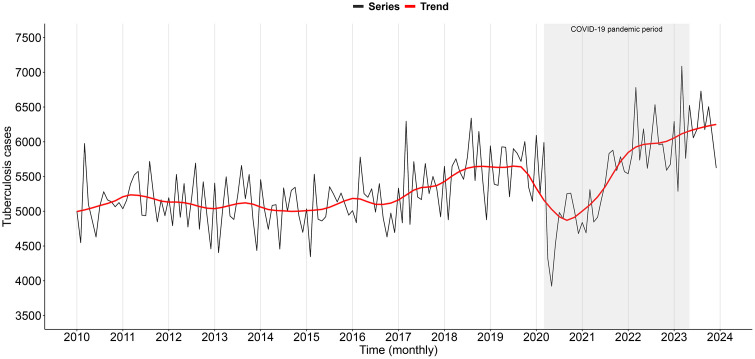
Monthly time series and temporal trend of tuberculosis cases in Brazil, 2010–2023. Source: Prepared by the authors.

Fig 1 shows the monthly counts of PTB cases, which display consistent seasonal variations over the years, with peaks occurring predominantly in March and August and sharper declines in February, June, and December. A downward trend emerged in late 2019 and intensified in March 2020, coinciding with the onset of the COVID-19 pandemic’s impact. Case numbers recovered gradually from the last quarter of 2020, reaching the average 2019 level only in October 2021. This pattern was similar across all five regions of the country (S3 Fig in S1 File).

The mean PTB incidence rate during the study period was 30.3 cases per 100,000 inhabitants-years (ranging from 31.2 in 2010 to 33.6 per 100,000 in 2023). Overall, the long-term trend was stable (AAPC = 0.87; 95% CI: −0.04 to 1.34). However, a significant upward trend was observed between 2021 and 2023 (APC = 7.34; 95% CI: 0.58 to 11.79), consistent with the temporal pattern in Fig 1. Across the entire period, increasing trends were documented among men (AAPC = 1.34; 95% CI: 0.81 to 1.78) and in the 20–29-year age group (AAPC = 2.22; 95% CI: 1.11 to 3.32) (Table 1).

**Table 1:** Temporal trend analysis of pulmonary tuberculosis cases according to epidemiological variables in Brazil, 2010-2023.

Variables	Rate^a^			Segmented period				Entire period		
	2010	2023	2010-2023	Period	APC	95% CI	Trend	AAPC	95% CI	Trend
Brazil	31.2	33.6	30.3	2010–2021	−0.32	−1.90 to 0.20	Stable	0.87	−0.04 to 1.34	Stable
				2021–2023	7.34^b^	0.58 to 11.79	Increasing			
Sex										
Male	42.9	49.8	43.0	2010–2013	−2.42^b^	−6.03 to −0.20	Decreasing	1.34^b^	0.81 to 1.78	Increasing
				2013–2018	2.22^b^	1.41 to 4.75	Increasing			
				2018–2021	−3.94^b^	−5.67 to −1.78	Decreasing			
				2021–2023	13.71^b^	9.31 to 17.23	Increasing			
Female	20.1	18.9	18.2	2010–2021	−1.33^b^	−3.50 to −0.84	Descending	0.01	−0.87 to 0.38	Stable
				2021–2023	7.67^b^	0.40 to 10.84	Increasing			
Age group (years)										
0–9	3.6	5.5	3.7	2010–2021	−0.98	−9.88 to 1.57	Stable	3.44	−0.21 to 5.38	Stable
				2021–2023	31.60^b^	2.11 to 49.76	Increasing			
10–19	12.7	15.9	14.0	2010–2023	0.62	−0.30 to 1.56	Stable	0.62	−0.30 to 1.56	Stable
20–29	42.6	49.8	44.1	2010–2023	2.22^b^	1.11 to 3.32	Increasing	2.22^b^	1.11 to 3.32	Increasing
30–39	42.2	43.6	38.1	2010–2020	−1.64^b^	−2.55 to −1.02	Decreasing	0.29	−0.44 to 0.97	Stable
				2020–2023	6.99^b^	2.36 to 14.79	Increasing			
40–49	45.6	40.6	37.9	2010–2013	8.67^b^	−18.07 to −0.88	Descending	−1.88^b^	−3.03 to −0.16	Decreasing
				2013–2023	0.25	−2.11 to 6.89	Stable			
50–59	38.1	41.0	39.7	2010–2023	−1.20^b^	−2.03 to −0.41	Decreasing	−1.20 ^b^	−2.03 to −0.41	Decreasing
60–69	41.2	32.1	35.9	2010–2023	−1.52^b^	−2.41 to −0.65	Decreasing	−1.52^b^	−2.41 to −0.65	Decreasing
70–79	41.1	35.9	34.1	2010–2020	−3.16^b^	−5.15 to −2.20	Decreasing	−1.00^b^	−2.18 to −0.08	Decreasing
				2020–2023	6.51^b^	0.12 to 16.36	Increasing			
≥ 80	35.0	32.5	29.4	2010–2023	−1.03	−2.30 to 0.15	Stable	−1.03	−2.30 to 0.15	Stable
Region/State										
North	39.8	53.3	43.7	2010–2015	−0.07	−3.89 to 1.50	Stable	2.24^b^	1.77 to 2.77	Increasing
				2015–2023	3.72^b^	2.90 to 5.52	Increasing			
Rondônia	25.5	34.4	29.1	2010–2012	8.74^b^	1.78 to 13.75	Increasing	1.90^b^	0.91 to 2.59	Increasing
				2012–2021	−1.45^b^	−3.92 to −0.86	Decreasing			
				2021–2023	10.97^b^	2.41 to 15.6	Increasing			
Acre	36.4	53.6	45.8	2010–2016	0.47	−1.88 to 1.78	Stable	2.56	1.75 to 3.10	Increasing
				2016–2020	8.58^b^	5.98 to 12.34	Increasing			
				2020–2023	−0.97	−7.93 to 2.10	Stable			
Amazonas	57.7	79.3	64.4	2010–2023	2.61^b^	1.78 to 3.44	Increasing	2.61^b^	1.78 to 3.44	Increasing
Roraima	25.5	69.6	38.0	2010–2015	−4.39	−11.7 to 0.52	Stable	7.35^b^	5.85 to 8.81	Increasing
				2015–2023	15.42^b^	12.46 to 19.39	Increasing			
Pará	41.5	51.4	43.9	2010–2014	−2.96^b^	−6.52 to −0.79	Decreasing	1.56^b^	1.01 to 2.08	Increasing
				2014–2023	3.64^b^	2.91 to 4.59	Increasing			
Amapá	25.2	42.4	30.0	2010–2014	−6.56	−19.71 to 0.57	Stable	3.68^b^	2.16 to 5.59	Increasing
				2014–2023	8.58^b^	6.24 to 12.6	Increasing			
Tocantins	12.4	12.8	11.5	2010–2016	−2.72	−12.5 to 0.23	Stable	0.61	−0.68 to 1.86	Stable
				2016–2023	3.56^b^	0.99 to 11.81	Increasing			
Northeast	32.1	32.2	29.6	2010–2015	−2.67^b^	−4.41 to −1.85	Decreasing	0.17	−0.24 to 0.49	Stable
				2015–2018	2.13^b^	0.30 to 3.40	Increasing			
				2018–2021	−2.56^b^	−3.99 to −0.87	Decreasing			
				2021–2023	8.99^b^	5.67 to 11.80	Increasing			
Maranhão	29.2	35.7	28.7	2010–2014	−4.71^b^	−9.92 to −1.54	Decreasing	0.99^b^	0.30 to 1.76	Increasing
				2014–2023	3.63^b^	2.59 to 5.04	Increasing			
Piauí	21.3	21.3	18.4	2010–2015	−7.55^b^	−17.29 to −3.39	Decreasing	−1.24	−2.45 to 0.15	Stable
				2015–2023	2.92^b^	0.68 to 8.17	Increasing			
Ceará	37.3	29.6	32.9	2010–2023	−0.79	2.61 to −0.99	Stable	−0.79	2.61 to −0.99	Stable
Rio Grande do Norte	24.7	29.3	29.0	2010–2015	−1.80^b^	−5.72 to −0.04	Decreasing	1.41^b^	0.39 to 1.92	Increasing
				2015–2018	10.44^b^	5.10 to 13.50	Increasing			
				2018–2023	−0.50	−5.58 to 1.32	Stable			
Paraíba	23.6	27.6	23.7	2010–2023	0.54	−1.33 to 2.40	Stable	0.54	−1.33 to 2.40	Stable
Pernambuco	41.3	49.1	41.7	2010–2021	−0.39	−2.03 to 0.26	Stable	1.28^b^	0.12 to 1.83	Increasing
				2021–2023	11.08^b^	1.60 to 15.50	Increasing			
Alagoas	28.8	28.1	27.0	2010–2018	−0.87	−1.66 to 1.92	Stable	−0.20	−1.16 to 0.60	Stable
				2018–2021	−7.10^b^	−7.10 to −10.01	Decreasing			
				2021–2023	14.16^b^	4.42 to 21.24	Increasing			
Sergipe	20.6	36.1	28.6	2010–2018	5.41^b^	4.77 to 6.47	Increasing	4.98^b^	4.13 to 5.42	Increasing
				2018–2021	−1.29	−3.40 to 2.04	Stable			
				2021–2023	13.24^b^	6.16 to 18.02	Increasing			
Bahia	33.3	26.4	25.3	2010–2023	−2.54^b^	−4.04 to −1.11	Decreasing	−2.54^b^	−4.04 to −1.11	Decreasing
Southeast	32.4	35.1	31.6	2010–2023	0.15	−0.77 to 1.08	Stable	0.15	−0.77 to 1.08	Stable
Minas Gerais	16.2	15.7	14.5	2010–2015	−3.72^b^	−6.16 to −2.61	Decreasing	−0.17	−0.74 to 0.20	Stable
				2015–2018	1.58	−1.09 to 3.02	Stable			
				2018–2021	−3.12^b^	−4.89 to −0.81	Decreasing			
				2021–2023	11.40^b^	6.51 to 14.99	Increasing			
Espírito Santo	31.9	34.5	27.5	2010–2016	−4.59^b^	−6.95 to −2.81	Decreasing	0.35	−0.35 to 1.04	Stable
				2016–2023	4.80^b^	3.23 to 6.83	Increasing			
Rio de Janeiro	61.0	62.5	55.1	2010–2014	−4.82^b^	−10.62 to −1.92	Decreasing	0.02	−1.08 to 0.84	Stable
				2014–2018	5.23^b^	2.64 to 10.02	Increasing			
				2018–2021	−7.86^b^	−11.18 to −3.88	Decreasing			
				2021–2023	12.83^b^	3.93 to 20.21	Increasing			
São Paulo	29.4	33.9	30.4	2010–2016	2.88^b^	1.59 to 4.86	Increasing	2.07^b^	1.20 to 2.66	Increasing
				2016–2021	−3.96^b^	−8.00 to −2.26	Decreasing			
				2021–2023	16.06^b^	6.90 to 21.66	Increasing			
South	27.0	25.8	25.1	2010–2023	−0.46	−1.41 to 0.45	Stable	−0.46	−1.41 to 0.45	Stable
Paraná	19.1	17.6	16.9	2010–2023	−0.71	−1.88 to 0.44	Stable	−0.71	−1.88 to 0.44	Stable
Santa Catarina	22.7	23.3	21.1	2010–2021	−1.95^b^	−5.63 to −0.66	Decreasing	0.18	−1.53 to 1.10	Stable
				2021–2023	12.76	−0.34 to 20.20	Stable			
Rio Grande do Sul	37.2	35.4	35.8	2010–2023	0.02	−0.86 to 0.88	Stable	0.02	−0.86 to 0.88	Stable
Central−West	19.4	23.1	20.2	2010–2023	0.25	−0.68 to 1.17	Stable	0.25	−0.68 to 1.17	Stable
Mato Grosso do Sul	29.2	49.9	34.2	2010–2015	−2.51	−12.01 to 1.43	Stable	2.83^b^	1.60 to 4.21	Increasing
				2015–2023	6.31^b^	4.24 to 11.48	Increasing			
Mato Grosso	34.3	29.5	32.5	2010–2023	−2.72^b^	−5.14 to −0.29	Decreasing	−2.72^b^	−5.14 to −0.29	Decreasing
Goiás	12.6	13.5	12.6	2010–2018	0.31	−0.01 to 1.79	Stable	0.89^b^	0.41 to 1.22	Increasing
				2018–2021	−1.93^b^	−3.35 to −0.37	Decreasing			
				2021–2023	7.73^b^	3.32 to 10.61	Increasing			
Distrito Federal	8.2	12.3	9.7	2010–2018	2.17^b^	0.99 to 5.47	Increasing	2.58^b^	1.01 to 3.65	Increasing
				2018–2021	−7.00^b^	−11.03 to −1.10	Decreasing			
				2021–2023	20.74^b^	5.53 to 31.21	Increasing			

*Abbreviations:* APC, annual percentage change; AAPC, average annual percent change; CI, confidence interval.

^a^Incidence rate per 100,000 inhabitants-years.

^b^Statistically significant (*p*-value < 0.05).

The North Region registered the highest incidence (43.7 cases per 100,000) and was the only region to show an increase trend (AAPC = 2.24; 95% CI: 1.77 to 2.77). Nevertheless, more than two-thirds of all reported cases were concentrated in the Southeast (45.1%; n = 403,730) and Northeast (26.5%; n = 237,448) regions (S1 Table in S1 File).

At the state level, the highest incidence rates were observed in Amazonas (64.4 per 100,000), Rio de Janeiro (55.1 per 100,000), Acre (45.8 per 100,000), Pará (43.9 per 100,000), and Pernambuco (41.7 per 100,000). Together, these five states accounted for almost one-third (32.3%; n = 289,546) of all cases reported during the study period (S4 Fig in S1 File). An increasing temporal trend was detected in 13 states and the Federal District—six in the North, four in the Northeast, three in the Central-West (two states plus the Federal District), and one in the Southeast. Bahia (Northeast) and Mato Grosso (Central-West) were the only states to exhibit decreasing trends over the period (AAPC = −2.54; 95% CI: −4.04 to −1.11 and AAPC = −2.72; 95% CI: −5.14 to −0.29, respectively). All other states showed stationary trends (Table 1).

### Spatial patterns

**Fig 2:**
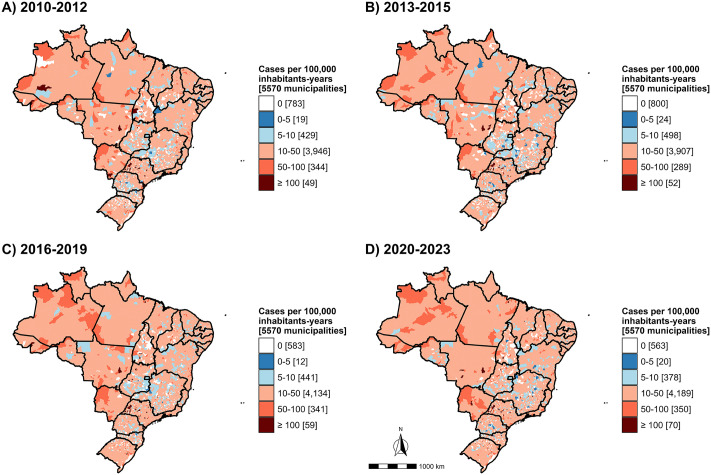
Incidence rate of pulmonary tuberculosis case in Brazil, 2010-2023. Source: Brazilian Institute of Geography and Statistics (IBGE), municipal territorial mesh (*Malha Municipal*).

Between the initial (2010–2012) and final (2020–2023) study periods, the number of municipalities with PTB incidence rates ≥ 100 cases per 100,000 inhabitants-years increased by 42.8%, rising from 49 to 70. Most of these municipalities were concentrated in the Southeast Region (57.1%, n = 40), mainly in São Paulo (n = 29) and Minas Gerais (n = 10), and in the South Region (15.7%, n = 11), with Rio Grande do Sul accounting for eight of them (Fig 2).

**Fig 3:**
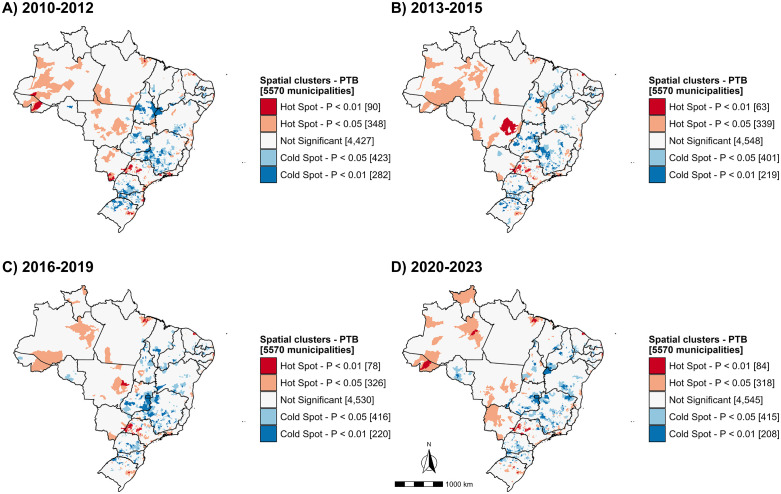
Spatial patterns of pulmonary tuberculosis cases using Local Getis-Ord *G*_i_^*^ statistic in Brazil, 2010-2023. Source: Brazilian Institute of Geography and Statistics (IBGE), municipal territorial mesh (*Malha Municipal*).

In addition, significant positive spatial autocorrelation was detected for 2010–2023 (Moran’s I = 0.233; p = 0.001), and this pattern persisted across all four study subperiods (S2 Table in S1 File). Fig 3 presents the results of the local spatial autocorrelation analysis, highlighting hot and cold spots identified with the Getis-Ord local *G*_i_^*^ statistic.

In total, 804 municipalities were classified as hotspots between 2010 and 2023, of which 168 (20.9%) persisted throughout every study period; these were located chiefly in the Southeast (49.4%) and North (17.3%) regions. Over time, the number of hotspot municipalities decreased in most regions of the country, except in the North, where the proportion rose from 11.6% (51/438) in 2010–2012 to 20.9% (84/402) in 2020–2023. The largest increases were observed in the states of Roraima (200%), Acre (180%), Amazonas (100%), and Pará (35.7%). Growth in the number of hotspots was also noted in Pernambuco (Northeast), Minas Gerais (Southeast), and Rio Grande do Sul (South). Notably, none of the study periods showed coldspots in Amazonas, Roraima, or Rio de Janeiro, whereas the Federal District exhibited no hotspots at any time (S3 Table in S1 File).

### Spatiotemporal clustering analysis

**Fig 4:**
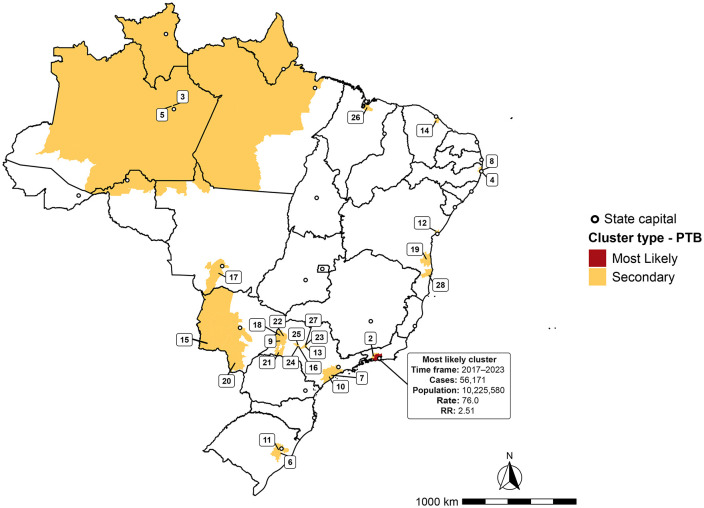
Spatiotemporal risk clusters for the occurrence of pulmonary tuberculosis in Brazil, 2010–2023. Source: Brazilian Institute of Geography and Statistics (IBGE), municipal territorial mesh (*Malha Municipal*).

The space–time scan statistic identified one most likely cluster and 27 secondary high-risk clusters for PTB transmission in Brazil (Fig 4 and Table 2). Together, these clusters encompassed 379 municipalities, fewer than half of all hotspot municipalities detected (47.1%, 379/804), and were located mainly in the North (42%, n = 159) and Southeast (26.4%, n = 100) regions.

**Table 2:** Spatiotemporal clusters of pulmonary tuberculosis incidence in Brazil, 2010-2023.

Cluster	Time frame	Center/ radius (km)	State	Number of municipalities	Observed	Expected	Rate^a^	RR	LLR	p-value
1	2017–2023	Rio de Janeiro/ 32.17	Rio de Janeiro	10	56171	23225.7	76.0	2.51	17291.4	<0.001
2	2010–2015	Rio de Janeiro/ 40.5	Rio de Janeiro	15	44517	19628.4	71.2	2.33	11922.9	<0.001
3	2017–2023	Manaus/ 1540.72	Amapá, Amazonas, Mato Grosso, Pará, Rondônia, Roraima	161	47186	23673.9	62.6	2.05	9353.0	<0.001
4	2010–2016	Recife/ 27.9	Pernambuco	10	17481	7484.5	73.4	2.36	4888.7	<0.001
5	2010–2015	Manaus/ 1540.72	Amapá, Amazonas, Mato Grosso, Pará, Rondônia, Roraima	161	30345	17592.5	54.2	1.75	3883.4	<0.001
6	2010–2016	Porto Alegre/ 64.85	Rio Grande do Sul	22	14416	6405.3	70.7	2.27	3719.9	<0.001
7	2017–2023	Peruíbe/ 86.9	São Paulo	36	59826	42469.0	44.3	1.44	3321.0	<0.001
8	2018–2022	Recife/ 27.9	Pernambuco	10	12416	5600.7	69.6	2.23	3095.2	<0.001
9	2012–2018	Presidente Prudente/ 48.69	São Paulo	25	2928	703.4	130.8	4.17	1953.8	<0.001
10	2010–2015	Peruíbe/ 86.9	São Paulo	36	46217	34572.0	42.0	1.36	1851.0	<0.001
11	2018–2022	Porto Alegre/ 54.53	Rio Grande do Sul	23	9966	5140.3	60.9	1.95	1785.5	<0.001
12	2010–2016	Salvador/ 20.32	Bahia	5	11553	6747.3	53.8	1.72	1420.5	<0.001
13	2012–2018	Bariri/ 15.76	São Paulo	4	760	96.2	248.2	7.91	907.4	<0.001
14	2010–2013	Itaitinga/ 23.72	Ceará	9	6592	3799.8	54.5	1.74	843.8	<0.001
15	2018–2023	Porto Murtinho/ 328.47	Mato Grosso do Sul	33	5279	3650.1	45.4	1.45	320.5	<0.001
16	2013–2019	Guaimbê/ 26.55	São Paulo	4	345	62.9	172.4	5.49	305.3	<0.001
17	2016–2022	Nossa Sra do Livramento/ 115.61	Mato Grosso	7	3373	2207.7	48.0	1.53	265.1	<0.001
18	2020–2022	Andradina/ 46.46	São Paulo	15	653	242.6	84.6	2.69	236.2	<0.001
19	2016–2022	São José da Vitória/ 45.72	Bahia	14	1991	1239.4	50.5	1.61	192.5	<0.001
20	2010–2016	Dourados/ 73.12	Mato Grosso do Sul	9	667	280.1	74.8	2.38	191.9	<0.001
21	2012–2018	Presidente Prudente/ 37.75	São Paulo	12	709	322.6	69.0	2.20	172.0	<0.001
22	2010–2010	Araçatuba/ 12.77	São Paulo	3	151	21.6	220.1	7.01	164.5	<0.001
23	2020–2022	Jaú/ 15.76	São Paulo	4	179	41.4	135.9	4.33	124.6	<0.001
24	2010–2011	Lins/ 26.55	São Paulo	4	84	17.4	151.3	4.81	65.5	<0.001
25	2021–2022	Lins/ 26.55	São Paulo	4	78	18.1	135.5	4.31	54.1	<0.001
26	2015–2015	São Luís/ 43.16	Maranhão	8	633	420.0	47.3	1.51	46.7	<0.001
27	2010–2010	Bariri/ 15.76	São Paulo	4	59	13.3	139.0	4.43	42.1	<0.001
28	2014–2016	Santa Cruz Cabrália/ 43.92	Bahia	3	245	143.0	53.8	1.71	29.9	<0.001

*Abbreviations:* km, kilometers; LLR, log-likelihood ratios; RR, relative risk.

^a^ Incidence rate adjusted by sex and age group per 100,000 inhabitants-years.

The most likely cluster was detected in the Rio de Janeiro Metropolitan Region (RJMR), covering ten municipalities—Nilópolis, Queimados, Belford Roxo, Rio de Janeiro, Duque de Caxias, São João de Meriti, Japeri, Nova Iguaçu, Mesquita, and Magé—between 2017 and 2023. During this period, 56,171 PTB cases (12% of the national total) were reported, yielding an adjusted incidence of 76 per 100,000 inhabitants-years and a RR of 2.51. Notably, the same area, expanded to include five additional RJMR municipalities (Miguel Pereira, Paracambi, Seropédica, Itaguaí, and Engenheiro Paulo de Frontin), was classified as the primary secondary cluster for 2010–2015, with an adjusted incidence of 71.2 per 100,000 inhabitants-years and an RR of 2.33. Also, in the Southeast Region, twelve clusters were located in the state of São Paulo, spanning 85 municipalities, the largest number for any single state, and accounting for 111,989 PTB cases (12.5% of all cases from 2010 to 2023), with RRs ranging from 1.44 to 7.91.

Clusters 3 and 5 comprised 161 municipalities distributed across six states of the Brazilian Legal Amazon. Their combined RR increased from 1.75 in 2010–2015 to 2.05 in 2017–2023. In the Northeast Region, clusters 4 and 8 were situated in ten municipalities of the Recife Metropolitan Region, Pernambuco, during 2010–2016 (RR = 2.36) and 2018–2022 (RR = 2.23), respectively.

Other secondary clusters included cluster 26 in the São Luís Metropolitan Region (RR = 1.51) in 2015; cluster 14 in the Fortaleza Metropolitan Region (RR = 1.74) for 2010–2013; cluster 12 in the Salvador Metropolitan Region (RR = 1.72) for 2010–2016; cluster 19 in southern Bahia (RR = 1.61) for 2016–2022; and cluster 28 in extreme southern Bahia (RR = 1.71) for 2014–2016.

In the South Region, clusters 6 and 11, both within the Porto Alegre Metropolitan Region, covered 25 municipalities during 2010–2016 (RR = 2.27) and 2018–2022 (RR = 1.95), respectively. In the Central-West Region, clusters 15 and 20, both in Mato Grosso do Sul, spanned 37 municipalities during 2018–2023 (RR = 1.45) and 2010–2016 (RR = 2.38), respectively.

**Fig 5:**
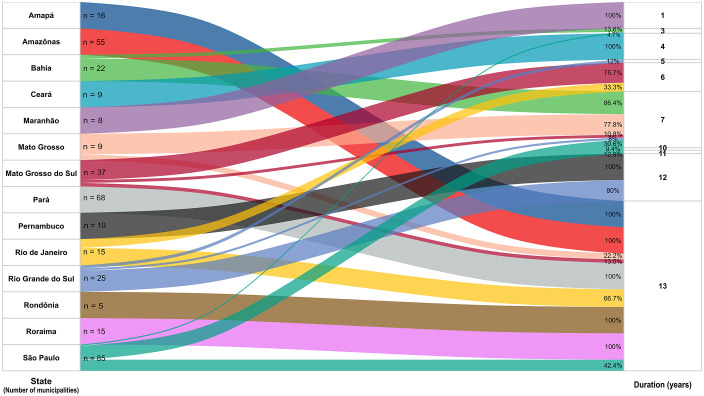
Flow chart showing the distribution of 379 municipalities by state, classified according to the duration of their status as spatiotemporal risk clusters for pulmonary tuberculosis in Brazil, 2010–2023. Source: Prepared by the authors.

Overall, 212 municipalities remained high-risk clusters for nearly the entire study period and were thus classified as high-priority areas. These municipalities were concentrated mainly in Pará (68), Amazonas (55), São Paulo (36), Amapá (16), Roraima (15), Rio de Janeiro (10), Rondônia (5), Mato Grosso do Sul (5), and Mato Grosso (2) (Fig 5). The complete list is provided in S4 Table in S1 File.

### Analysis of spatial variation in temporal trends

**Fig 6:**
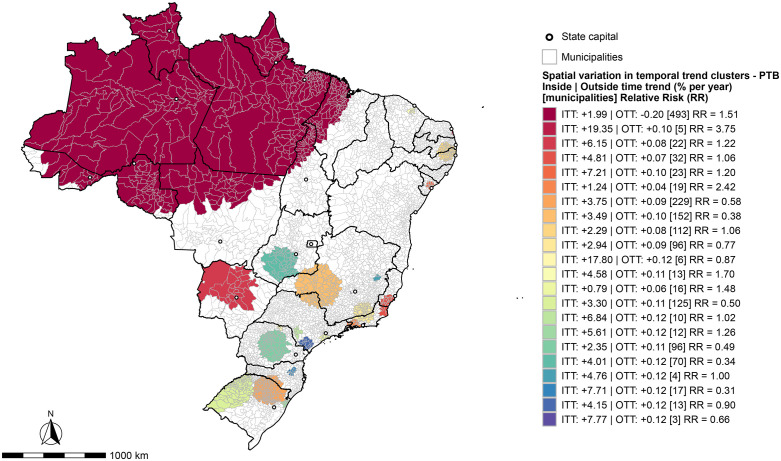
Spatial variation in temporal trends clusters for the occurrence of pulmonary tuberculosis in Brazil, 2010-2023. Source: Brazilian Institute of Geography and Statistics (IBGE), municipal territorial mesh (*Malha Municipal*).

The SVTT analysis indicated an average annual increase of 0.122% in the PTB incidence rate between 2010 and 2023, reflecting near-stationary growth, consistent with the overall trend shown in Table 1. The scan further identified one most likely cluster and 21 secondary clusters (Fig 6 and S5 Table in S1 File).

The primary cluster included 493 municipalities located across all states of the Legal Amazon. This cluster exhibited an ITT of 1.99% growth per year, whereas the OTT showed a slight decline of −0.20% per year, with a RR of 1.51. The primary secondary cluster was detected in the state of Rio Grande do Norte and included the municipalities of Vila Flor, Senador Georgino Avelino, Nísia Floresta, Arês, and Tibau do Sul. In this cluster, the ITT was 19.35% per year, while the OTT indicated a modest increase of 0.10% per year, with an RR of 3.75. Fig 6 presents the remaining secondary clusters identified in the study, all of which showed increasing trends in both ITT and OTT and were distributed across the various regions of Brazil.

## Discussion

Our study identified critical areas of PTB distribution in Brazil at the municipal level and examined their temporal evolution from 2010 to 2023. We found consistent seasonal variations in monthly case counts throughout the study period. Peaks occurred chiefly in March, followed by August and, to a lesser extent, October, months that coincide with increased demand for health services after vacations, recesses, or surveillance campaigns. The sharpest declines were recorded in February, June, and December, periods traditionally marked by extended holidays, reduced clinic hours, or lower health-service utilization. This regularity suggests seasonality linked to diagnostic opportunity, underscoring the need for surveillance strategies that account for this pattern to optimize TB detection and control [30].

A downturn in case notifications began in the last quarter of 2019 and deepened in March 2020 with the onset of the COVID-19 pandemic. Public fear of visiting health facilities, social-distancing measures, and the diversion of resources to the COVID-19 response markedly reduced the demand for diagnosis and treatment of other diseases, including TB [31]. Partial suspension of TB surveillance and control activities, interruption of screening campaigns, and limited availability of laboratory tests further contributed to underreporting and a temporary weakening of the TB response, explaining the abrupt decline in notifications during 2020 [32].

Consequently, the PTB incidence rate, previously high but relatively stable, fell sharply between 2020 and 2021, returning to pre-pandemic levels only in 2022. Despite this rebound, the results indicate that, although progress has been made in prevention, diagnosis, and treatment, the decline in TB incidence remains insufficient for Brazil to meet the targets set by the World Health Organization (WHO) and by the National Plan to End TB as a Public Health Problem by 2030 [33]. Consistent with these results, a nationwide Bayesian age–period–cohort analysis suggests that PTB incidence in Brazil is unlikely to decline substantially by 2035. Incidence is projected to increase or remain persistently high in metropolitan areas, particularly among men, while non-metropolitan areas are expected to stabilize at levels still far from WHO elimination targets [10].

A recent study partly supports our findings, showing that most TB program indicators in Brazil, including preventive treatment, directly observed therapy, and contact investigation, remain below the 90% coverage targets established by the WHO for 2030 [33]. According to that study, TB incidence in Brazil could reach 38.8 per 100,000 inhabitants by 2025, well above the WHO target of 16.7. To achieve the goals, the authors highlight the need to strengthen program management, address key TB determinants, and intensify integrated, coordinated interventions. Complementing these recommendations, another study projects that public policies aimed at reducing the prison population could lower TB incidence by roughly 16.4% (95% CI: 11.4–23.3) by 2034, representing an important opportunity to reignite progress toward disease elimination [34].

In the same direction, recent evidence shows that scaling up screening and preventive treatment among priority populations in Brazil could prevent approximately 15% (95% CI: 12.8–17.5%) of all TB episodes by 2050 and avert an estimated 1.8 million DALYs (95% CI: 1.2–2.3) over the period. Although expanding these interventions would require up to 62% of Brazil’s national TB budget in 2030, costs would gradually decline and remain well below cost-effectiveness thresholds. Notably, the study estimates that every dollar invested would return roughly 51 dollars to society, reinforcing that preventive interventions are highly cost-effective and yield substantial social, economic, and health benefits [35].

The increasing trend in PTB rates among men and adults aged 20–29 may reflect a combination of factors: greater exposure to high-risk environments, such as precarious workplaces and crowded households, high mobility and economic activity typical of this age group, and barriers to timely health care access that often delay diagnosis and adequate treatment [36,37]. Alcohol and drug use, social vulnerability, and the high TB burden in prisons exacerbate the situation [34,38]. COVID-19-related service disruptions may also have aggravated these inequities and diagnosis delays, partly explaining the upturn observed between 2021 and 2023 [31].

Complementarily, the increasing trends observed in certain states and regions after 2014 may be associated with fiscal austerity policies that gradually weakened public health financing and reduced the responsiveness of the SUS [39]. As noted, these vulnerabilities were further aggravated during the COVID-19 pandemic. Moreover, political instability and the dismantling of health governance structures in recent years, characterized by successive changes in the Ministry of Health leadership and the circulation of anti-scientific rhetoric, further undermined the capacity of the system to meet population needs. This context had a particularly strong impact in historically vulnerable regions such as the Amazon, where health-system collapse became an imminent risk. Recent evidence supports this interpretation, demonstrating that political denialism contributed to increased mortality, declining vaccination coverage, and the weakening of Brazil’s National Immunization Program [40], factors that can also directly affect TB control by undermining essential surveillance, prevention, and continuity-of-care efforts.

Given the influence of time on disease distribution, we applied a space–time scan analysis to complement the spatial assessment. This approach identified 15 municipalities in the RJMR as the most likely high-risk cluster for nearly the entire study period, along with 27 secondary clusters throughout the country. The largest secondary cluster comprised 161 municipalities in the Legal Amazon and remained high-risk for 13 years, indicating extensive areas where the PTB transmission chain persists. These areas should therefore be prioritized in upcoming TB control and prevention strategies.

The findings for the RJMR had already been documented in a previous study, that reported an RR of 2.68 in 25 municipalities between 2001 and 2008 [5]. The RJMR houses one of Brazil’s largest urban populations and exhibits a combination of structural and social factors that facilitate PTB transmission. High population density, especially in informal settlements such as *favelas*, combined with intense mobility and social inequality creates a favorable environment for disease spread [41]. The region also has historically high TB/HIV coinfection rates [2] and drug-resistant TB rates [42], substantial homeless populations [43], and a large incarcerated population [44], all of which are recognized TB risk factors. These characteristics make TB control in the RJMR a persistent challenge, emphasizing the need for intersectoral, place-based interventions.

Although this study did not directly assess the role of prisons in TB transmission, it is essential to acknowledge that incarceration remains a major structural driver of TB in Brazil [45]. Evidence demonstrated that incarcerated individuals experience extremely high TB incidence, that transmission networks link prisons and surrounding municipalities, and that these patterns are influenced by social inequality and mobility of the incarcerated population [8,46]. Future studies could build upon our findings by incorporating incarceration-related indicators to further clarify the spatial interplay between institutional and community transmission.

The spatial and temporal heterogeneity observed in Brazil parallels patterns reported in other high TB burden countries in Latin America, such as Peru, and Venezuela where socioeconomic vulnerability, overcrowding, and fragmented health systems similarly sustain persistent transmission [1,47]. These findings reinforce that structural inequities, including poverty, housing precarity, incarceration, and migration, are regional determinants of TB, and that spatially targeted, intersectoral interventions are crucial for accelerating progress toward the End TB targets across Latin America [34,48].

Municipalities in the Legal Amazon likewise warrant priority attention in TB control policies, particularly due to persistent adverse socioeconomic indicators that favor transmission [36]. The situation is even more complex in Indigenous communities, where vulnerability stems not only from poverty but also from broader structural factors such as limited land access, territorial insecurity, and climate-change effects [49,50]. These structural vulnerabilities may also interact with environmental stressors, including the effects of climate variability, which have recently been recognized as emerging determinants of TB dynamics [50].

In addition, informal and unsafe economic activities, such as illegal mining (*garimpos*), deforestation-related labor, and seasonal agricultural work, remain widespread in parts of the Amazon and Central-West regions [51]. These contexts often involve precarious or forced labor conditions, population mobility, and intense exposure to silica dust and smoke from forest fires, all of which may amplify TB vulnerability through both social and biological pathways [52]. Addressing these socioenvironmental determinants is therefore essential for developing integrated TB control strategies adapted to these frontier territories.

Furthermore, the North Region has been the main entry point for Venezuelan migration since 2016, with flows intensifying after 2018 as the economic crisis in Venezuela deepened. This sudden population influx into already fragile areas worsens housing and sanitary conditions, raising PTB transmission risk [53]. Added to this are the logistical challenges typical of the Amazon—vast distances, low population density, and long travel times to health centers—that hamper timely diagnosis and treatment [54,55].

Decentralizing diagnosis and ensuring treatment within primary care, tailored to the sociocultural contexts of Indigenous peoples and refugees, is therefore essential for strengthening TB control nationwide, particularly in the Legal Amazon [36,53,54]. Yet a prior study indicated that the North has the nation’s lowest coverage of the GeneXpert MTB/RIF test, a key tool for rapid, accurate TB diagnosis and rifampicin resistance detection [56]. Scarcity of this resource impedes early case detection and timely treatment initiation, especially in remote, vulnerable Amazonian areas.

In São Paulo State, 12 spatiotemporal PTB risk clusters were detected. Among them, clusters 7 and 10, located in the São Paulo and Baixada Santista metropolitan regions, are characterized by high population density, rapid urbanization, pockets of poverty, and pronounced social inequalities [37,57]. These findings reveal ongoing zones of active TB transmission in critical urban settings where precarious housing, overcrowding, intense mobility, and access barriers to regular health care sustain the transmission chain [5–7,37]. São Paulo, Brazil’s most populous state and a major internal and international migration hub, also faces complex population dynamics that demand robust, localized responses [58]. Strengthening local surveillance, expanding active case-finding, promoting early diagnosis, and targeting interventions that address social determinants of health are crucial for mitigating the structural inequities underlying TB burden in the state [57].

Additional spatiotemporal PTB clusters were identified in Maranhão, Pernambuco, and Bahia (Northeast) and in Rio Grande do Sul (South), all near their respective metropolitan regions. These results underscore not only the wide spatial and temporal spread of TB but also the need for coordinated public policies across states, especially in densely populated urban areas [10,37].

The SVTT analysis yielded worrisome findings, indicating that without effective interventions in the high-risk areas identified, a substantial rise in PTB incidence is likely in the coming years, particularly in the large cluster encompassing all states of the Legal Amazon. Projected growth in these territories indicates expanding risk areas, including locations not previously flagged as critical. These data point to a worsening epidemiological scenario and call for urgent action by public-health authorities.

### Limitations and strengths

Our study has several limitations. First, the analyses relied on secondary data, which are subject to under-reporting, reporting delays, and inconsistencies, particularly in remote areas. Nonetheless, the quality of TB records in Brazil has improved substantially in recent years [59]. Second, because more disaggregated data, such as residential addresses, were unavailable, all analyses were conducted at the municipal level, which may limit the precise identification of local transmission foci. However, this level of aggregation allows for a broader, more comprehensive assessment, supporting the identification of priority territories for intervention and reducing the loss of generalizability that can occur with highly disaggregated data. Third, the space–time scan statistic is sensitive to the definition of spatial and temporal scanning windows, which may influence cluster size and boundaries [26]. Furthermore, although sex and age were included as covariates, the method cannot fully capture relevant contextual determinants, such as internal migration flows, population mobility, barriers to health-care access, and local environmental or structural factors, that also influence disease dynamics. Future studies should integrate such contextual variables to provide a more comprehensive, place-based understanding of TB persistence across territories.

Fourth, as an ecological study, the analyses are subject to ecological bias, as associations observed at the municipal level may not reflect individual-level relationships. We also acknowledge the modifiable areal unit problem, whereby results can vary depending on the spatial scale or zoning scheme used, potentially influencing the comparability and interpretation of spatial patterns. In addition, changes in surveillance sensitivity over time may have influenced some of the temporal trends we observed; we could not clearly disentangle the extent to which those trends reflect true changes in disease risk versus fluctuations in case-detection capacity. Finally, the persistence of certain clusters over time may point to entrenched social determinants that are difficult to address, posing an ongoing challenge for TB control in Brazil.

Despite these limitations, the study has important strengths. Foremost is the use of a robust, integrated methodological framework that combines temporal trend analysis, global and local spatial autocorrelation, and space–time scanning. This approach allowed us not only to pinpoint areas with high disease concentration but also to assess their persistence and evolution over time. The broad territorial coverage and inclusion of recent data provide an up-to-date view of PTB distribution in Brazil and highlight critical areas—such as the Rio de Janeiro Metropolitan Region, municipalities in the Legal Amazon, and several in São Paulo State—that warrant priority attention from TB control programs. In addition, the findings are likely applicable to settings with similar socioeconomic and demographic characteristics, particularly in Latin America, thereby informing regional TB-control strategies.

Another strength lies in the practical utility of the results, which can guide the planning and implementation of more effective, locally tailored surveillance and control measures. Finally, the study offers valuable evidence for anticipating future trends, showing that PTB is likely to increase in historically vulnerable areas unless specific, coordinated interventions are adopted.

### Recommendations and public health implications

Urgent enhancements in TB surveillance and diagnostic capacity are critical, especially through the deployment of rapid molecular testing and the decentralization of services in identified high-risk municipalities, remote areas, and Indigenous communities. Given the widespread and entrenched nature of PTB in Brazil, isolated measures will be insufficient. Instead, multi-sectoral strategies must coordinate health care with social assistance, housing, sanitation, and justice services, embedding social-protection policies and the guarantee of basic rights at every level, overall in the population living in vulnerable settings. Finally, sustained, integrated monitoring is essential to track evolving hotspots and gauge the impact of control strategies across Brazil.

## Conclusion

This study provides the most comprehensive spatiotemporal assessment of PTB in Brazil to date, covering municipal-level data from 2010 to 2023. By integrating time-series, spatial autocorrelation, and space–time scan analyses, it identified persistent and expanding high-risk clusters, particularly in RJMR, in the Legal Amazon, and major urban centers.

The study’s main contribution lies in revealing how these long-term spatial–temporal dynamics expose structural vulnerabilities that sustain TB transmission, offering actionable evidence for territorial health planning. The identification of 212 municipalities as high-priority for public-health interventions enables more precise prioritization of surveillance, diagnostic expansion, and resource allocation within the national TB control strategy.

These findings underscore the need for structured, regionalized, and intersectoral responses focused on strengthening primary care, decentralizing diagnosis, providing timely and adequate treatment, and reducing social inequalities. Importantly, they reaffirm that TB is not only a biomedical issue but also a social and territorial process shaped by the organization of space and the unequal conditions under which people live. Therefore, without sustained, coordinated efforts, Brazil cannot realistically achieve the WHO’s target of eliminating TB as a public-health problem by 2030.

## Supporting information

S1 File Supplementary material containing additional figures, tables, and descriptive analyses. (DOCX)

## References

[1] World Health Organization. Global tuberculosis report 2024. Geneva: WHO; 2024.

[2] Ministério da Saúde do Brasil. Boletim epidemiológico - tuberculose 2025. Brasília: Distrito Federal: Ministério da Saúde; 2025.

[3] Migliori GB, Dowdy D, Denholm JT, D’Ambrosio L, Centis R. The path to tuberculosis elimination: a renewed vision. Eur Respir J. 2023;61(6):2300499. doi: 10.1183/13993003.00499-2023 37080572

[4] Liu M-Y, Li Q-H, Zhang Y-J, Ma Y, Liu Y, Feng W, et al. Spatial and temporal clustering analysis of tuberculosis in the mainland of China at the prefecture level, 2005-2015. Infect Dis Poverty. 2018;7(1):106. doi: 10.1186/s40249-018-0490-8 30340513 PMC6195697

[5] de Paiva JPS, Magalhães MAFM, Leal TC, da Silva LF, da Silva LG, do Carmo RF, et al. Time trend, social vulnerability, and identification of risk areas for tuberculosis in Brazil: An ecological study. PLoS One. 2022;17(1):e0247894. doi: 10.1371/journal.pone.0247894 35077447 PMC8789117

[6] Harling G, Castro MC. A spatial analysis of social and economic determinants of tuberculosis in Brazil. Health Place. 2014;25:56–67. doi: 10.1016/j.healthplace.2013.10.008 24269879

[7] Berra TZ, Ramos ACV, Arroyo LH, Delpino FM, de Almeida Crispim J, Alves YM, et al. Risk-prone territories for spreading tuberculosis, temporal trends and their determinants in a high burden city from São Paulo State, Brazil. BMC Infect Dis. 2022;22(1):515. doi: 10.1186/s12879-022-07500-5 35655177 PMC9161466

[8] Pelissari DM, Diaz-Quijano FA. Impact of incarceration on tuberculosis incidence and its interaction with income distribution inequality in Brazil. Trans R Soc Trop Med Hyg. 2020;114(1):23–30. doi: 10.1093/trstmh/trz088 31667507

[9] Jesus GS, Gestal PFPS, Silva AF, Cavalcanti DM, Lua I, Ichihara MY, et al. Effects of conditional cash transfers on tuberculosis incidence and mortality according to race, ethnicity and socioeconomic factors in the 100 Million Brazilian Cohort. Nat Med. 2025;31(2):653–62. doi: 10.1038/s41591-024-03381-0 39753969 PMC11835739

[10] da Silva JMN, Diaz-Quijano FA, Sanchez MN, Ramalho WM. Projecting tuberculosis control progress in metropolitan and non-metropolitan areas of Brazil, 2001–2035: a Bayesian age-period-cohort analysis. Infect Dis Poverty. 2025;14(1):125. doi: 10.1186/s40249-025-01400-x 41430721 PMC12720469

[11] Zille AI, Werneck GL, Luiz RR, Conde MB. Social determinants of pulmonary tuberculosis in Brazil: an ecological study. BMC Pulm Med. 2019;19(1):87. doi: 10.1186/s12890-019-0855-1 31068155 PMC6507035

[12] Shaweno D, Karmakar M, Alene KA, Ragonnet R, Clements AC, Trauer JM, et al. Methods used in the spatial analysis of tuberculosis epidemiology: a systematic review. BMC Med. 2018;16(1):193. doi: 10.1186/s12916-018-1178-4 30333043 PMC6193308

[13] Zhang Y, Ye J, Hou S, Lu X, Yang C, Pi Q, et al. Spatial-temporal analysis of pulmonary tuberculosis in Hubei Province, China, 2011-2021. PLoS One. 2023;18(2):e0281479. doi: 10.1371/journal.pone.0281479 36749779 PMC9904469

[14] Instituto Brasileiro de Geografia e Estatística. Brasil em síntese. 2022 [cited 5 May 2025]. Available from: https://brasilemsintese.ibge.gov.br/

[15] Instituto Brasileiro de Geografia e Estatística. Amazônia Legal. [cited 5 May 2025]. Available from: https://www.ibge.gov.br/geociencias/cartas-e-mapas/mapas-regionais/15819-amazonia-legal.html?lang=pt-BR&t=o-que-e

[16] Rocha MS, Bartholomay P, Cavalcante MV, Medeiros FCD, Codenotti SB, Pelissari DM, et al. Sistema de Informação de Agravos de Notificação (Sinan): principais características da notificação e da análise de dados relacionada à tuberculose. Epidemiol Serv Saude. 2020;29(1):e2019017. doi: 10.5123/S1679-49742020000100009 32074197

[17] Dong Y, Peng C-YJ. Principled missing data methods for researchers. Springerplus. 2013;2(1):222. doi: 10.1186/2193-1801-2-222 23853744 PMC3701793

[18] Rubin DB. Multiple Imputation for Nonresponse in Surveys. 1st ed. Wiley; 1987. doi: 10.1002/9780470316696

[19] Madley-Dowd P, Hughes R, Tilling K, Heron J. The proportion of missing data should not be used to guide decisions on multiple imputation. J Clin Epidemiol. 2019;110:63–73. doi: 10.1016/j.jclinepi.2019.02.016 30878639 PMC6547017

[20] Cleveland RB, Cleveland WS, Irma T. STL: A seasonal-trend decomposition procedure based on LOESS. J Off Stat. 1990;6:3–73.

[21] Kim H-J, Chen H-S, Byrne J, Wheeler B, Feuer EJ. Twenty years since Joinpoint 1.0: two major enhancements, their justification, and impact. Stat Med. 2022;41(16):3102–30. doi: 10.1002/sim.9407 35522060

[22] Anselin L. Local Indicators of Spatial Association—LISA. Geogr Anal. 1995;27(2):93–115. doi: 10.1111/j.1538-4632.1995.tb00338.x

[23] Yu Y, Wu B, Wu C, Wang Q, Hu D, Chen W. Spatial-temporal analysis of tuberculosis in Chongqing, China 2011-2018. BMC Infect Dis. 2020;20(1):531. doi: 10.1186/s12879-020-05249-3 32698763 PMC7374877

[24] Getis A, Ord JK. The analysis of spatial association by use of distance statistics. Geogr Anal. 1992;24(3):189–206. doi: 10.1111/j.1538-4632.1992.tb00261.x

[25] Kulldorff M, Nagarwalla N. Spatial disease clusters: detection and inference. Stat Med. 1995;14(8):799–810. doi: 10.1002/sim.4780140809 7644860

[26] Kulldorf M. SaTScan user guide. 2022. Available from: https://www.satscan.org/techdoc.html

[27] Waller LA, Gotway CA. Applied spatial statistics for public health data. 1st ed. Wiley; 2004. doi: 10.1002/0471662682

[28] Kulldorff M, Athas WF, Feurer EJ, Miller BA, Key CR. Evaluating cluster alarms: a space-time scan statistic and brain cancer in Los Alamos, New Mexico. Am J Public Health. 1998;88(9):1377–80. doi: 10.2105/ajph.88.9.1377 9736881 PMC1509064

[29] Huang L, Tiwari RC, Zou Z, Kulldorff M, Feuer EJ. Weighted normal spatial scan statistic for heterogeneous population data. J Am Stat Assoc. 2009;104(487):886–98. doi: 10.1198/jasa.2009.ap07613

[30] Paz LC, Saavedra CAPB, Braga JU, Kimura H, Evangelista MDSN. Analysis of the seasonality of tuberculosis in Brazilian capitals and the Federal District from 2001 to 2019. Cad Saude Publica. 2022;38(7):e00291321. doi: 10.1590/0102-311XPT291321 35894370

[31] Souza MDR, da Paz WS, Sales VBDS, de Jesus GFH, Tavares DDS, Lima SVMA, et al. Impact of the COVID-19 pandemic on the diagnosis of tuberculosis in Brazil: is the WHO end TB strategy at risk? Front Pharmacol. 2022;13:891711. doi: 10.3389/fphar.2022.891711 35847020 PMC9277074

[32] Berra TZ, Ramos ACV, Alves YM, Tavares RBV, Tartaro AF, Nascimento MCD, et al. Impact of COVID-19 on tuberculosis indicators in Brazil: a time series and spatial analysis study. Trop Med Infect Dis. 2022;7(9):247. doi: 10.3390/tropicalmed7090247 36136658 PMC9500936

[33] Villalva-Serra K, Barreto-Duarte B, Rodrigues MM, Queiroz ATL, Martinez L, Croda J, et al. Impact of strategic public health interventions to reduce tuberculosis incidence in Brazil: a Bayesian structural time-series scenario analysis. Lancet Reg Health Am. 2024;41:100963. doi: 10.1016/j.lana.2024.100963 39759249 PMC11697790

[34] Liu YE, Mabene Y, Camelo S, Rueda ZV, Pelissari DM, Dockhorn Costa Johansen F, et al. Mass incarceration as a driver of the tuberculosis epidemic in Latin America and projected effects of policy alternatives: a mathematical modelling study. Lancet Public Health. 2024;9(11):e841–51. doi: 10.1016/S2468-2667(24)00192-0 39419058 PMC11602220

[35] Vesga JF, Mohamed MS, Shandal M, Jabbour E, Lomtadze N, Kubjane M, et al. The effectiveness, cost-effectiveness, budget impact, and return on investment of scaling up tuberculosis screening and preventive treatment in Brazil, Georgia, Kenya, and South Africa: a modelling study. Lancet Glob Health. 2025;13(11):e1857–68. doi: 10.1016/S2214-109X(25)00321-3 41109257

[36] de Castro DB, de Seixas Maciel EMG, Sadahiro M, Pinto RC, de Albuquerque BC, Braga JU. Tuberculosis incidence inequalities and its social determinants in Manaus from 2007 to 2016. Int J Equity Health. 2018;17(1):187. doi: 10.1186/s12939-018-0900-3 30594205 PMC6310934

[37] Pelissari DM, Rocha MS, Bartholomay P, Sanchez MN, Duarte EC, Arakaki-Sanchez D, et al. Identifying socioeconomic, epidemiological and operational scenarios for tuberculosis control in Brazil: an ecological study. BMJ Open. 2018;8(6):e018545. doi: 10.1136/bmjopen-2017-018545 29880560 PMC6009496

[38] Silva DR, Muñoz-Torrico M, Duarte R, Galvão T, Bonini EH, Arbex FF, et al. Risk factors for tuberculosis: diabetes, smoking, alcohol use, and the use of other drugs. J Bras Pneumol. 2018;44(2):145–52. doi: 10.1590/s1806-37562017000000443 29791552 PMC6044656

[39] Maia LR, Campos MR, Castanheira D. Fiscal austerity and municipal health spending: an interrupted time series study. Rev Saude Publica. 2024;58:42. doi: 10.11606/s1518-8787.2024058005772 39319934 PMC11548907

[40] Razafindrakoto M, Roubaud F, Castilho MR, Pero V, Saboia J. Investigating the “Bolsonaro effect” on the spread of the Covid-19 pandemic: an empirical analysis of observational data in Brazil. PLoS One. 2024;19(4):e0288894. doi: 10.1371/journal.pone.0288894 38635577 PMC11025779

[41] Pereira AGL, Medronho R de A, Escosteguy CC, Valencia LIO, Magalhães M de AFM. Spatial distribution and socioeconomic context of tuberculosis in Rio de Janeiro, Brazil. Rev Saude Publica. 2015;49:48. doi: 10.1590/S0034-8910.2015049005470 26270014 PMC4544397

[42] Pungartnik PC, Viana PVDS, Santos JPCD, Macedo LR, Berra TZ, Paiva NS. Spatial analysis of drug resistant tuberculosis (DRTB) incidence and relationships with determinants in Rio de Janeiro state, 2010 to 2022. PLoS One. 2025;20(5):e0321553. doi: 10.1371/journal.pone.0321553 40315278 PMC12047809

[43] Gioseffi JR, Brignol SMS, Werneck GL. Sociodemographic profile of homeless people notified with tuberculosis in the municipality of Rio de Janeiro, Brazil, from 2015 to 2019. Cad Saude Publica. 2023;39(10):e00051122. doi: 10.1590/0102-311XPT051122 37851728 PMC10581682

[44] Sánchez A, Larouzé B. Tuberculosis control in prisons, from research to action: the Rio de Janeiro, Brazil, experience. Cien Saude Colet. 2016;21(7):2071–80. doi: 10.1590/1413-81232015217.08182016 27383341

[45] Cords O, Martinez L, Warren JL, O’Marr JM, Walter KS, Cohen T, et al. Incidence and prevalence of tuberculosis in incarcerated populations: a systematic review and meta-analysis. Lancet Public Health. 2021;6(5):e300–8. doi: 10.1016/S2468-2667(21)00025-6 33765455 PMC8168455

[46] Walter KS, Dos Santos PCP, Gonçalves TO, da Silva BO, da Silva Santos A, de Cássia Leite A, et al. The role of prisons in disseminating tuberculosis in Brazil: A genomic epidemiology study. Lancet Reg Health Am. 2022;9:100186. doi: 10.1016/j.lana.2022.100186 35647574 PMC9140320

[47] Ranzani OT, Pescarini JM, Martinez L, Garcia-Basteiro AL. Increasing tuberculosis burden in Latin America: an alarming trend for global control efforts. BMJ Glob Health. 2021;6(3):e005639. doi: 10.1136/bmjgh-2021-005639 33762254 PMC7993346

[48] Litvinjenko S, Magwood O, Wu S, Wei X. Burden of tuberculosis among vulnerable populations worldwide: an overview of systematic reviews. Lancet Infect Dis. 2023;23(12):1395–407. doi: 10.1016/S1473-3099(23)00372-9 37696278 PMC10665202

[49] Polidoro M, de Oliveira DC. Prevalence and spatial autocorrelation of tuberculosis in indigenous people in Brazil, 2002-2022. J Racial Ethn Health Disparities. 2025;12(2):865–72. doi: 10.1007/s40615-024-01924-2 38300427

[50] Saunders MJ, Boccia D, Khan PY, Goscé L, Gasparrini A, Clark RA, et al. Climate change and tuberculosis: an analytical framework. Lancet Respir Med. 2025. doi: 10.1016/S2213-2600(25)00329-7 41177169 PMC7618504

[51] Cortinhas Ferreira Neto L, Diniz CG, Maretto RV, Persello C, Silva Pinheiro ML, Castro MC, et al. Uncontrolled illegal mining and garimpo in the Brazilian Amazon. Nat Commun. 2024;15(1):9847. doi: 10.1038/s41467-024-54220-2 39537611 PMC11560919

[52] Ellwanger JH, Kulmann-Leal B, Kaminski VL, Valverde-Villegas JM, Veiga ABGD, Spilki FR, et al. Beyond diversity loss and climate change: Impacts of Amazon deforestation on infectious diseases and public health. An Acad Bras Cienc. 2020;92(1):e20191375. doi: 10.1590/0001-3765202020191375 32321030

[53] Alves YM, Berra TZ, Tavares RBV, Popolin MAP, da Costa FBP, de Jezus SV, et al. International migration, refugees, and spread of tuberculosis in Brazil: analysis of clusters, trends, and associated factors (2010-2021). Trop Med Infect Dis. 2024;9(4):82. doi: 10.3390/tropicalmed9040082 38668543 PMC11053982

[54] Mesquita CR, da Conceição ML, de Oliveira RAC, Conceição EC, Garcez JCD, Sousa IFR, et al. Spatial analysis of tuberculosis patient flow in a neglected region of Northern Brazil. Trop Med Infect Dis. 2023;8(8):397. doi: 10.3390/tropicalmed8080397 37624335 PMC10457907

[55] Garrido MDS, Bührer-Sékula S, Souza ABD, Ramasawmy R, Quincó PDL, Monte RL, et al. Temporal distribution of tuberculosis in the State of Amazonas, Brazil. Rev Soc Bras Med Trop. 2015;48 Suppl 1:63–9. doi: 10.1590/0037-8682-0055-2014 26061372

[56] Aguilar-Jiménez JR, Pelissari DM, Diaz-Quijano FA. How has the municipal availability of the GeneXpert®MTB/RIF system affected the detection of drug-resistant tuberculosis in Brazil? Trop Med Int Health. 2024;29(1):57–62. doi: 10.1111/tmi.13945 37919228

[57] da Silva JMN, Diaz-Quijano FA. The yield of tuberculosis contact investigation in São Paulo, Brazil: a community-based cross-sectional study. Epidemiol Infect. 2025;153:e20. doi: 10.1017/S0950268824001675 39881574 PMC11795444

[58] de Almeida Soares D, Placeres A, Arcêncio RA, Fronteira I. Evidence on tuberculosis in migrants at Brazil’s international borders: a scoping review. J Migr Health. 2023;7:100167. doi: 10.1016/j.jmh.2023.100167 36846810 PMC9945690

[59] Bartholomay P, Oliveira GPD, Pinheiro RS, Vasconcelos AMN. Melhoria da qualidade das informações sobre tuberculose a partir do relacionamento entre bases de dados. Cad Saúde Pública. 2014;30:2459–70. doi: 10.1590/0102-311X00116313 25493999

