# Supplementary material for: Space-time clustering and temporal trend analysis of pulmonary tuberculosis in Brazil, 2010–2023
Source: PLoS One. 2026 Jan 8;21(1):e0339784. doi: 10.1371/journal.pone.0339784 (PMC12782390; doi:10.1371/journal.pone.0339784)
Supplement: S1 File — (DOCX) [file pone.0339784.s001.docx]

**Supplemental Material**

**Space-time clustering and temporal trend analysis of pulmonary tuberculosis in Brazil, 2010–2023**

**Table of Contents**

**S1 Fig.** Map of Brazil and its territorial divisions………………………………………………2

**S1 Table.** Epidemiological characteristics of pulmonary tuberculosis cases in Brazil, 2010–2023…………………………………………………………………………………………….3

**S2 Fig.** Monthly time series and temporal trends of tuberculosis cases in Brazil by region, 2010–2023….…………………………….…………………………………………………………...8

**S3 Fig.** Heatmap of reported pulmonary tuberculosis cases across different states in Brazil, 2010-2023..…………………………………………………………………………………….9

**S4 Table.** Global spatial autocorrelation analysis of pulmonary tuberculosis notification rate in Brazil, 2010-2023……..………………………………………………………………………10

**S3 Table.** Descriptive analysis of significant spatial clusters of hot spots and cold spots of pulmonary tuberculosis cases by region and state in Brazil, 2010–2023………………………11

**S4 Table.** Distribution of 379 municipalities by region and state, according to the duration of classification as spatiotemporal risk clusters for pulmonary tuberculosis in Brazil, 2010–2023…………………………………………………………………………………………...14

**S5 Table.** Spatial variation in temporal trends clusters for the occurrence of pulmonary tuberculosis in Brazil, 2010-2023.….…………………………………………………………25

**
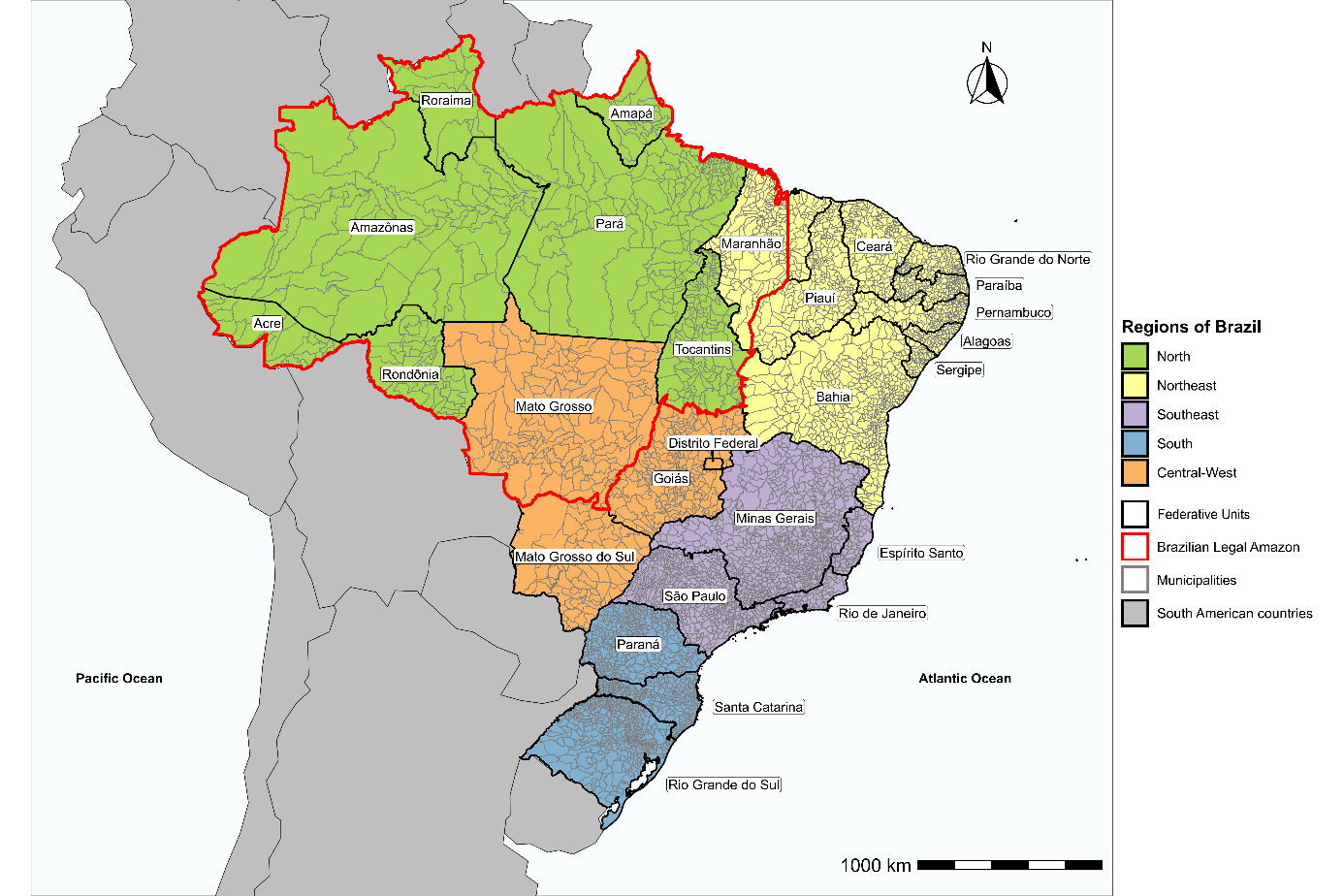
**

**S1 Fig.** Map of Brazil and its territorial divisions.

Source: Brazilian Institute of Geography and Statistics (IBGE), municipal territorial mesh (Malha Municipal).

**Note:** The thematic maps were produced using official and publicly available territorial grid data provided by the IBGE, <https://www.ibge.gov.br/geociencias/organizacao-do-territorio/malhas-territoriais.html>. Additional cartographic layers were generated using Natural Earth public-domain datasets accessed via the R package *rnaturalearth*, licensed under the MIT License and suitable for scientific publication

TB patients notified and

registered in Sinan from 2010 to 2023

N=1,290,055

Transfer N=39,421

Recurrence N=93,976

Retreatment N=113,938

Ignored N=28

New cases of TB

N=1,042,692

Change of diagnosis N=8,783

Non-pulmonary tuberculosis N=137,847

896,062 new PTB cases

included in the study

**S2 Fig.** Selection of study population.

Source: Prepared by the authors.

| **S1 Table.** Epidemiological characteristics of pulmonary tuberculosis cases in Brazil, 2010–2023. | | |
| --- | --- | --- |
| **Variables** | **N** | **%** |
| **Total of PTB cases** | **896,062** | **100,0** |
| **Period of diagnosis** |  |  |
| 2010-2012 | 184,501 | 20.6 |
| 2013-2015 | 182,115 | 20.3 |
| 2016-2019 | 259,733 | 29.0 |
| 2020-2023 | 269,713 | 30.1 |
| **Area of residence** |  |  |
| North | 106,368 | 11.9 |
| Northeast | 237,448 | 26.5 |
| Southeast | 403,730 | 45.1 |
| South | 104,118 | 11.6 |
| Central-West | 44,230 | 4.9 |
| Ignored | 168 | <0.1 |
| **Age, years** |  |  |
| Mean (SD) | 39.9 | 17.5 |
| Median (IQR) | 37.0 | 26.0-52.0 |
| **Age group (years)** |  |  |
| 0-9 | 15,361 | 1.7 |
| 10-19 | 63,636 | 7.1 |
| 20-29 | 221,758 | 24.7 |
| 30-39 | 181,157 | 20.2 |
| 40-49 | 152,313 | 17.0 |
| 50-59 | 126,810 | 14.2 |
| 60-69 | 79,027 | 8.8 |
| 70-79 | 39,513 | 4.4 |
| ≥80 | 16,252 | 1.8 |
| Ignored | 235 | <0.1 |
| **Sex** |  |  |
| Male | 620,704 | 69.3 |
| Female | 275,282 | 30.7 |
| Ignored | 76 | <0.1 |
| **Self-reported race** |  |  |
| White | 263,299 | 29.4 |
| Brown or mixed | 426,619 | 47.6 |
| Black | 113,087 | 12.6 |
| Asian | 7,373 | 0.8 |
| Indigenous | 10,158 | 1.1 |
| Ignored | 75,526 | 8.4 |
| **Education (years)** |  |  |
| Illiterate | 97,388 | 10.9 |
| 1-3 | 104,916 | 11.7 |
| 4-7 | 207,393 | 23.1 |
| 8-11 | 237,758 | 26.5 |
| ≥12 | 45,977 | 5.1 |
| Ignored | 202,630 | 22.6 |
| **HIV status** |  |  |
| Negative | 618,464 | 69.0 |
| Positive | 76,369 | 8.5 |
| Unknown | 201,229 | 22.5 |
| **AIDS** |  |  |
| No | 712,558 | 79.5 |
| Yes | 68,284 | 7.6 |
| Ignored | 115,219 | 12.9 |
| **Alcohol user** |  |  |
| No | 673,017 | 75.1 |
| Yes | 152,258 | 17.0 |
| Ignored | 70,787 | 7.9 |
| **Smoking** |  |  |
| No | 470,703 | 52.5 |
| Yes | 148,336 | 16.6 |
| Ignored | 277,023 | 30.9 |
| **Drug user** |  |  |
| No | 524,124 | 58.5 |
| Yes | 92,105 | 10.3 |
| Ignored | 279,833 | 31.2 |
| **Diabetes mellitus** |  |  |
| No | 748,350 | 83.5 |
| Yes | 74,123 | 8.3 |
| Ignored | 73,589 | 8.2 |
| **Mental disorder** |  |  |
| No | 799,332 | 89.2 |
| Yes | 20,635 | 2.3 |
| Ignored | 76,095 | 8.5 |
| **Other comorbidities** |  |  |
| No | 477,984 | 53.3 |
| Yes | 79,898 | 8.9 |
| Ignored | 338,180 | 37.7 |
| **Bacteriological status** |  |  |
| Not confirmed | 243,639 | 27.2 |
| Confirmed | 652,423 | 72.8 |

*Note:* Self-reported race, classified as Branco (White), Preto (Black), Pardo (Brown or mixed), Amarelo (Asian) or Indígena (Indigenous).

*Abbreviations:* PTB, pulmonary tuberculosis; SD, standard deviation; IQR, interquartile range; **AIDS,** acquired immunodeficiency syndrome; HIV, human immunodeficiency virus.

**
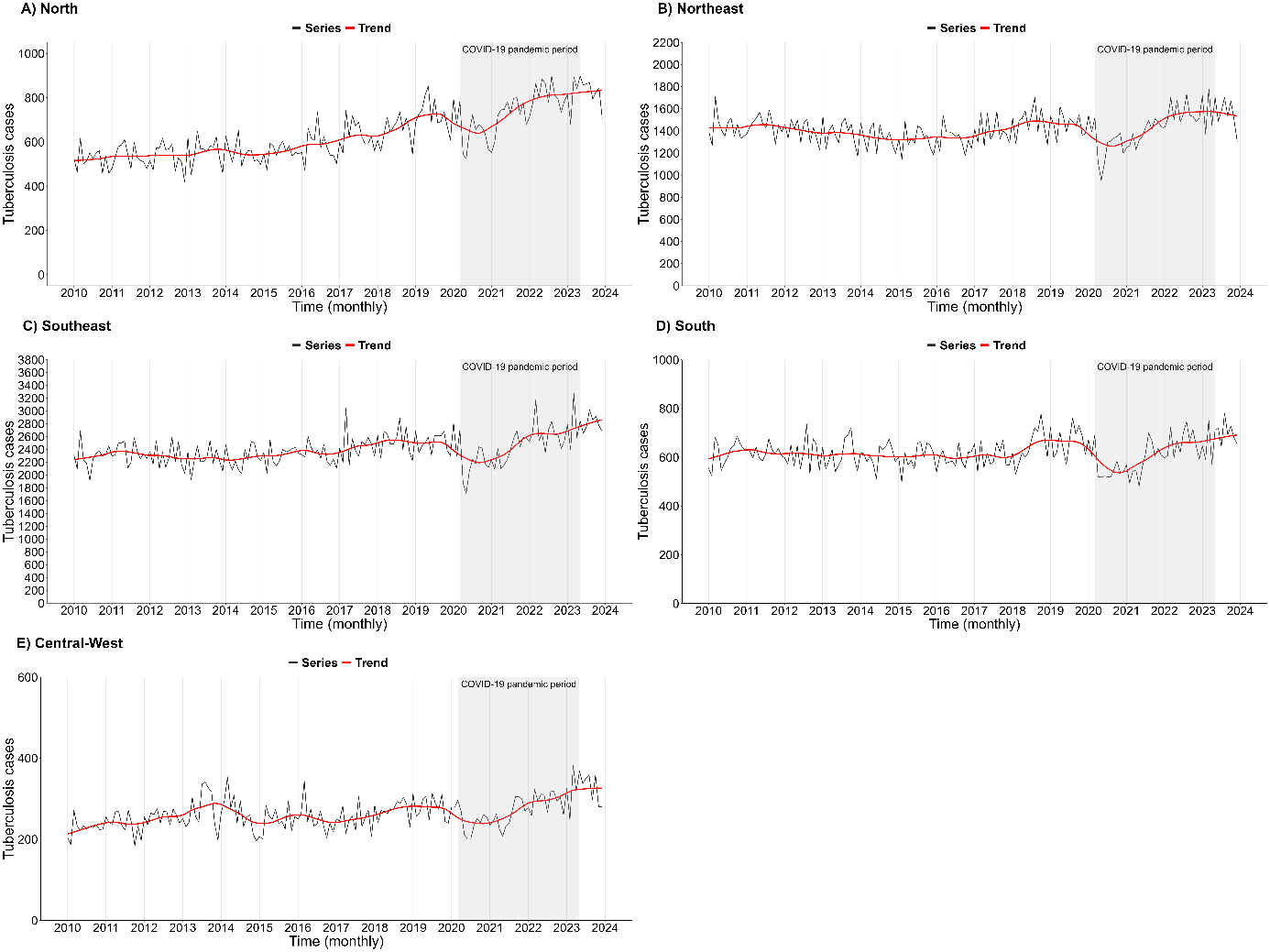
**

**S3 Fig.** Monthly time series and temporal trends of tuberculosis cases in Brazil by region, 2010–2023.

Source: Prepared by the authors.

**
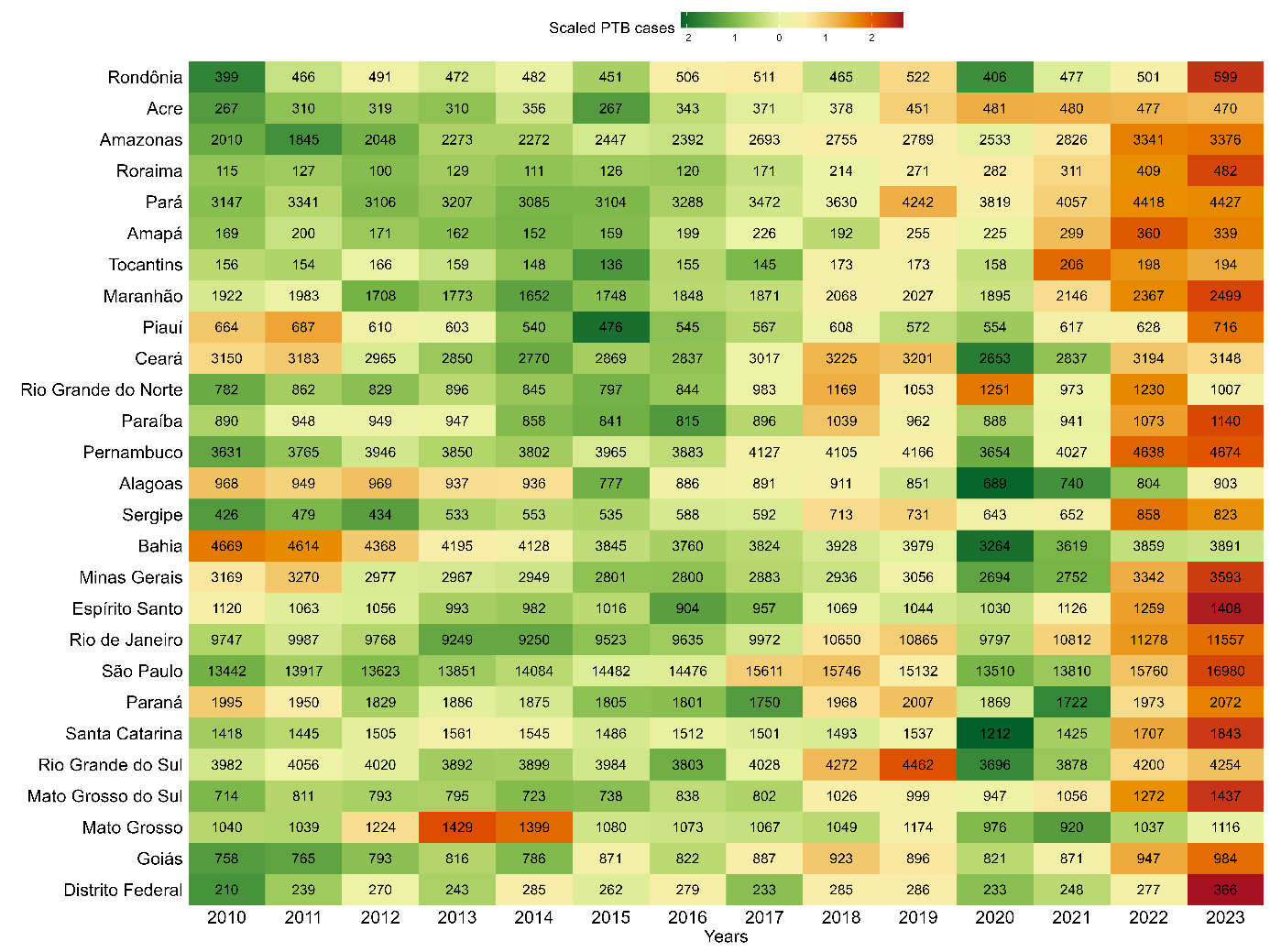
**

**S4 Fig.** Heatmap of reported pulmonary tuberculosis cases across different states in Brazil, 2010-2023.

Source: Prepared by the authors.

**S2 Table.** Global spatial autocorrelation analysis of pulmonary tuberculosis notification rate in Brazil, 2010-2023.

| **Period** | **Moran’s *I*** | ***Z*-score** | ***P*-value** | **Distribution pattern** |
| --- | --- | --- | --- | --- |
| **2010-2012** | 0.186 | 17.61 | 0.001 | Cluster |
| **2013-2015** | 0.157 | 15.65 | 0.001 | Cluster |
| **2016-2019** | 0.184 | 17.06 | 0.001 | Cluster |
| **2020-2023** | 0.165 | 17.10 | 0.001 | Cluster |
| **2010-2023** | 0.233 | 18.71 | 0.001 | Cluster |

| **S3 Table.** Descriptive analysis of significant spatial clusters of hot spots and cold spots of pulmonary tuberculosis cases by region and state in Brazil, 2010–2023. | | | | | | | | |
| --- | --- | --- | --- | --- | --- | --- | --- | --- |
| **Variables** | **Type of cluster** | | | | | | | |
|  | **Cold Spot**^a^ | | | | **Hot Spot**^a^ | | | |
|  | **2010-2012**  N = 705^b^ | **2013-2015**  N = 620^b^ | **2016-2019**  N = 636^b^ | **2020-2023**  N = 623^b^ | **2010-2012**  N = 438^b^ | **2013-2015**  N = 402^b^ | **2016-2019**  N = 404^b^ | **2020-2023**  N = 402^b^ |
| **Region/State** |  |  |  |  |  |  |  |  |
| North | 33 (4.7%) | 21 (3.4%) | 34 (5.3%) | 27 (4.3%) | 51 (11.6%) | 62 (15.4%) | 76 (18.8%) | 84 (20.9%) |
| Acre | 0 (0.0%) | 0 (0.0%) | 1 (0.2%) | 0 (0.0%) | 5 (1.1%) | 7 (1.7%) | 12 (3.0%) | 14 (3.5%) |
| Amazônas | - | - | - | - | 9 (2.1%) | 22 (5.5%) | 19 (4.7%) | 18 (4.5%) |
| Goiás | 57 (8.1%) | 59 (9.5%) | 74 (11.6%) | 41 (6.6%) | 7 (1.6%) | 3 (0.7%) | 6 (1.5%) | 4 (1.0%) |
| Pará | 0 (0.0%) | 0 (0.0%) | 4 (0.6%) | 2 (0.3%) | 32 (7.3%) | 28 (7.0%) | 37 (9.2%) | 38 (9.5%) |
| Rondônia | 1 (0.1%) | 0 (0.0%) | 4 (0.6%) | 10 (1.6%) | - | - | - | - |
| Roraima | - | - | - | - | 0 (0.0%) | 0 (0.0%) | 3 (0.7%) | 9 (2.2%) |
| Tocantins | 32 (4.5%) | 21 (3.4%) | 25 (3.9%) | 15 (2.4%) | 5 (1.1%) | 5 (1.2%) | 5 (1.2%) | 5 (1.2%) |
| Northeast | 92 (13.0%) | 102 (16.5%) | 141 (22.2%) | 141 (22.6%) | 97 (22.1%) | 64 (15.9%) | 60 (14.9%) | 56 (13.9%) |
| Alagoas | 0 (0.0%) | 3 (0.5%) | 1 (0.2%) | 4 (0.6%) | 10 (2.3%) | 7 (1.7%) | 3 (0.7%) | 0 (0.0%) |
| Bahia | 28 (4.0%) | 22 (3.5%) | 36 (5.7%) | 41 (6.6%) | 37 (8.4%) | 21 (5.2%) | 10 (2.5%) | 12 (3.0%) |
| Ceará | 2 (0.3%) | 1 (0.2%) | 8 (1.3%) | 14 (2.2%) | 27 (6.2%) | 12 (3.0%) | 11 (2.7%) | 11 (2.7%) |
| Maranhão | 5 (0.7%) | 20 (3.2%) | 14 (2.2%) | 11 (1.8%) | 1 (0.2%) | 1 (0.2%) | 0 (0.0%) | 4 (1.0%) |
| Paraíba | 23 (3.3%) | 26 (4.2%) | 31 (4.9%) | 8 (1.3%) | 0 (0.0%) | 3 (0.7%) | 0 (0.0%) | 1 (0.2%) |
| Pernambuco | 14 (2.0%) | 6 (1.0%) | 8 (1.3%) | 12 (1.9%) | 14 (3.2%) | 17 (4.2%) | 25 (6.2%) | 20 (5.0%) |
| Piauí | 12 (1.7%) | 21 (3.4%) | 18 (2.8%) | 35 (5.6%) | 5 (1.1%) | 0 (0.0%) | 0 (0.0%) | 0 (0.0%) |
| Rio Grande do Norte | 8 (1.1%) | 2 (0.3%) | 23 (3.6%) | 15 (2.4%) | 3 (0.7%) | 3 (0.7%) | 8 (2.0%) | 4 (1.0%) |
| Sergipe | 0 (0.0%) | 1 (0.2%) | 2 (0.3%) | 1 (0.2%) | 0 (0.0%) | 0 (0.0%) | 3 (0.7%) | 4 (1.0%) |
| Southeast | 241 (34.2%) | 194 (31.3%) | 234 (36.8%) | 223 (35.8%) | 163 (37.2%) | 180 (44.8%) | 177 (43.8%) | 155 (38.6%) |
| Espírito Santo | 0 (0.0%) | 4 (0.6%) | 5 (0.8%) | 8 (1.3%) | 3 (0.7%) | 0 (0.0%) | 0 (0.0%) | 4 (1.0%) |
| Minas Gerais | 207 (29.4%) | 166 (26.8%) | 195 (30.7%) | 175 (28.1%) | 20 (4.6%) | 23 (5.7%) | 8 (2.0%) | 33 (8.2%) |
| São Paulo | 34 (4.8%) | 24 (3.9%) | 34 (5.3%) | 40 (6.4%) | 108 (24.7%) | 130 (32.3%) | 143 (35.4%) | 91 (22.6%) |
| Rio de Janeiro | - | - | - | - | 32 (7.3%) | 27 (6.7%) | 26 (6.4%) | 27 (6.7%) |
| South | 280 (39.7%) | 241 (38.9%) | 149 (23.4%) | 187 (30.0%) | 82 (18.7%) | 58 (14.4%) | 62 (15.3%) | 68 (16.9%) |
| Paraná | 77 (10.9%) | 66 (10.6%) | 63 (9.9%) | 70 (11.2%) | 14 (3.2%) | 12 (3.0%) | 5 (1.2%) | 7 (1.7%) |
| Rio Grande do Sul | 103 (14.6%) | 94 (15.2%) | 45 (7.1%) | 53 (8.5%) | 44 (10.0%) | 33 (8.2%) | 51 (12.6%) | 60 (14.9%) |
| Santa Catarina | 100 (14.2%) | 81 (13.1%) | 41 (6.4%) | 64 (10.3%) | 24 (5.5%) | 13 (3.2%) | 6 (1.5%) | 1 (0.2%) |
| Central-West | 59 (8.4%) | 62 (10.0%) | 78 (12.3%) | 45 (7.2%) | 45 (10.3%) | 38 (9.5%) | 29 (7.2%) | 39 (9.7%) |
| Distrito Federal | 1 (0.1%) | 1 (0.2%) | 1 (0.2%) | 1 (0.2%) | - | - | - | - |
| Mato Grosso | 1 (0.1%) | 1 (0.2%) | 2 (0.3%) | 3 (0.5%) | 20 (4.6%) | 24 (6.0%) | 10 (2.5%) | 15 (3.7%) |
| Mato Grosso do Sul | 0 (0.0%) | 1 (0.2%) | 1 (0.2%) | 0 (0.0%) | 18 (4.1%) | 11 (2.7%) | 13 (3.2%) | 20 (5.0%) |
| ^a^ Hot spots and cold spots identified via Getis-Ord *Gi** (p < 0.05); ^b^ n (%) – municipalities | | | | | | | | |

| **S4 Table.** Distribution of 379 municipalities by region and state, according to the duration of classification as spatiotemporal risk clusters for pulmonary tuberculosis in Brazil, 2010–2023. | | | |
| --- | --- | --- | --- |
| **Region/State** | **Duration (years)** | **Cases** | **Rate^b^** |
| North – Amapá (n = 16) | | | |
| Amapá^a^ | 13 | 31 | 14.1 |
| Calçoene^a^ | 13 | 48 | 17.9 |
| Cutias^a^ | 13 | 16 | 12.7 |
| Ferreira Gomes^a^ | 13 | 13 | 7.6 |
| Itaubal^a^ | 13 | 11 | 8.3 |
| Laranjal do Jari^a^ | 13 | 207 | 19.6 |
| Macapá^a^ | 13 | 1806 | 15.4 |
| Mazagão^a^ | 13 | 61 | 11.5 |
| Oiapoque^a^ | 13 | 153 | 23.2 |
| Pedra Branca do Amapari^a^ | 13 | 39 | 12.2 |
| Porto Grande^a^ | 13 | 57 | 12.0 |
| Pracuúba^a^ | 13 | 6 | 5.7 |
| Santana^a^ | 13 | 374 | 12.8 |
| Serra do Navio^a^ | 13 | 9 | 7.3 |
| Tartarugalzinho^a^ | 13 | 31 | 8.8 |
| Vitória do Jari^a^ | 13 | 45 | 13.8 |
| North – Amazonas (n = 55) | | | |
| Alvarães^a^ | 13 | 28 | 6.9 |
| Amaturá^a^ | 13 | 45 | 16.5 |
| Anamã^a^ | 13 | 24 | 8.9 |
| Anori^a^ | 13 | 39 | 8.6 |
| Apuí^a^ | 13 | 49 | 9.4 |
| Autazes^a^ | 13 | 254 | 25.4 |
| Barcelos^a^ | 13 | 115 | 19.8 |
| Barreirinha^a^ | 13 | 127 | 15.8 |
| Benjamin Constant^a^ | 13 | 168 | 17.2 |
| Beruri^a^ | 13 | 71 | 14.6 |
| Boa Vista Do Ramos^a^ | 13 | 58 | 11.3 |
| Borba^a^ | 13 | 158 | 17.0 |
| Caapiranga^a^ | 13 | 47 | 14.2 |
| Canutama^a^ | 13 | 58 | 14.1 |
| Carauari^a^ | 13 | 93 | 12.4 |
| Careiro^a^ | 13 | 121 | 14.0 |
| Careiro da Várzea^a^ | 13 | 58 | 10.0 |
| Coari^a^ | 13 | 479 | 24.0 |
| Codajás^a^ | 13 | 77 | 12.3 |
| Fonte Boa^a^ | 13 | 65 | 9.8 |
| Humaitá^a^ | 13 | 277 | 20.2 |
| Iranduba^a^ | 13 | 454 | 33.1 |
| Itacoatiara^a^ | 13 | 596 | 22.8 |
| Itamarati^a^ | 13 | 66 | 25.9 |
| Itapiranga^a^ | 13 | 32 | 12.9 |
| Japurá^a^ | 13 | 14 | 6.5 |
| Juruá^a^ | 13 | 25 | 8.6 |
| Jutaí^a^ | 13 | 88 | 14.9 |
| Lábrea^a^ | 13 | 161 | 14.3 |
| Manacapuru^a^ | ‘13 | 849 | 33.2 |
| Manaquiri^a^ | 13 | 75 | 14.2 |
| Manaus^a^ | 13 | 23,486 | 43.8 |
| Manicoré^a^ | 13 | 211 | 15.5 |
| Maraã^a^ | 13 | 38 | 8.6 |
| Maués^a^ | 13 | 385 | 24.9 |
| Nhamundá^a^ | 13 | 82 | 15.9 |
| Nova Olinda do Norte^a^ | 13 | 175 | 22.4 |
| Novo Airão^a^ | 13 | 80 | 19.4 |
| Novo Aripuanã^a^ | 13 | 101 | 16.6 |
| Parintins^a^ | 13 | 571 | 21.0 |
| Presidente Figueiredo^a^ | 13 | 201 | 25.4 |
| Rio Preto da Eva^a^ | 13 | 142 | 20.9 |
| Santa Isabel do Rio Negro^a^ | 13 | 88 | 20.4 |
| Santo Antônio do Içá^a^ | 13 | 72 | 10.0 |
| Silves^a^ | 13 | 53 | 19.7 |
| São Gabriel Da Cachoeira^a^ | 13 | 472 | 39.2 |
| São Paulo de Olivença^a^ | 13 | 116 | 13.2 |
| São Sebastião do Uatumã^a^ | 13 | 42 | 13.9 |
| Tabatinga^a^ | 13 | 529 | 32.8 |
| Tapauá^a^ | 13 | 84 | 16.5 |
| Tefé^a^ | 13 | 600 | 32.6 |
| Tonantins^a^ | 13 | 80 | 16.4 |
| Uarini^a^ | 13 | 20 | 5.6 |
| Urucará^a^ | 13 | 57 | 11.8 |
| Urucurituba^a^ | 13 | 90 | 15.9 |
| North – Pará (n = 68) | | | |
| Abaetetuba^a^ | 13 | 551 | 13.5 |
| Afuá^a^ | 13 | 50 | 5.1 |
| Alenquer^a^ | 13 | 191 | 11.7 |
| Almeirim^a^ | 13 | 91 | 9.9 |
| Altamira^a^ | 13 | 551 | 18.1 |
| Anajás^a^ | 13 | 72 | 10.1 |
| Ananindeua^a^ | 13 | 4483 | 34.7 |
| Anapu^a^ | 13 | 58 | 8.3 |
| Aveiro^a^ | 13 | 21 | 4.6 |
| Bagre^a^ | 13 | 49 | 6.6 |
| Baião^a^ | 13 | 80 | 6.7 |
| Barcarena^a^ | 13 | 656 | 21.4 |
| Belterra^a^ | 13 | 61 | 13.2 |
| Belém^a^ | 13 | 15,888 | 42.9 |
| Benevides^a^ | 13 | 416 | 26.9 |
| Brasil Novo^a^ | 13 | 65 | 12.2 |
| Breves^a^ | 13 | 381 | 14.1 |
| Cachoeira do Arari^a^ | 13 | 57 | 9.6 |
| Cametá^a^ | 13 | 583 | 16.8 |
| Chaves^a^ | 13 | 24 | 4.3 |
| Colares^a^ | 13 | 72 | 22.0 |
| Curralinho^a^ | 13 | 63 | 7.4 |
| Curuá^a^ | 13 | 34 | 9.6 |
| Curuçá^a^ | 13 | 265 | 25.7 |
| Faro^a^ | 13 | 21 | 9.1 |
| Gurupá^a^ | 13 | 83 | 10.0 |
| Igarapé-Miri^a^ | 13 | 200 | 12.0 |
| Itaituba^a^ | 13 | 572 | 19.2 |
| Jacareacanga^a^ | 13 | 188 | 38.4 |
| Juruti^a^ | 13 | 177 | 13.3 |
| Limoeiro do Ajuru^a^ | 13 | 52 | 7.0 |
| Marituba^a^ | 13 | 1711 | 57.2 |
| Medicilândia^a^ | 13 | 50 | 6.8 |
| Melgaço^a^ | 13 | 52 | 7.3 |
| Mocajuba^a^ | 13 | 100 | 13.6 |
| Moju^a^ | 13 | 224 | 10.7 |
| Mojuí dos Campos^a^ | 13 | 21 | 4.4 |
| Monte Alegre^a^ | 13 | 89 | 5.7 |
| Muaná^a^ | 13 | 116 | 10.8 |
| Novo Progresso^a^ | 13 | 155 | 19.8 |
| Novo Repartimento^a^ | 13 | 112 | 6.6 |
| Oeiras do Pará^a^ | 13 | 91 | 10.7 |
| Oriximiná^a^ | 13 | 307 | 17.3 |
| Pacajá^a^ | 13 | 91 | 8.2 |
| Placas^a^ | 13 | 68 | 12.0 |
| Ponta de Pedras^a^ | 13 | 69 | 10.0 |
| Portel^a^ | 13 | 151 | 9.7 |
| Porto de Moz^a^ | 13 | 91 | 8.9 |
| Prainha^a^ | 13 | 38 | 4.3 |
| Rurópolis^a^ | 13 | 46 | 4.5 |
| Salvaterra^a^ | 13 | 95 | 16.1 |
| Santa Bárbara do Pará^a^ | 13 | 183 | 35.9 |
| Santa Cruz do Arari^a^ | 13 | 48 | 23.0 |
| Santa Izabel do Pará^a^ | 13 | 2084 | 116.9 |
| Santarém^a^ | 13 | 1340 | 16.1 |
| Santo Antônio do Tauá^a^ | 13 | 138 | 18.8 |
| Senador José Porfírio^a^ | 13 | 49 | 10.5 |
| Soure^a^ | 13 | 150 | 23.7 |
| São Caetano de Odivelas^a^ | 13 | 84 | 18.7 |
| São João da Ponta^a^ | 13 | 16 | 12.4 |
| São Sebastião da Boa Vista^a^ | 13 | 78 | 11.9 |
| Terra Santa^a^ | 13 | 67 | 14.0 |
| Trairão^a^ | 13 | 43 | 10.0 |
| Tucuruí^a^ | 13 | 559 | 21.5 |
| Uruará^a^ | 13 | 106 | 8.8 |
| Vigia^a^ | 13 | 335 | 25.1 |
| Vitória do Xingu^a^ | 13 | 80 | 20.6 |
| Óbidos^a^ | 13 | 153 | 11.1 |
| North – Rondônia (n = 5) | | | |
| Candeias do Jamari^a^ | 13 | 137 | 23.8 |
| Cujubim^a^ | 13 | 33 | 7.9 |
| Itapuã do Oeste^a^ | 13 | 47 | 20.1 |
| Machadinho D'oeste^a^ | 13 | 64 | 7.4 |
| Porto Velho^a^ | 13 | 3639 | 29.4 |
| North – Roraima (n = 15) | | | |
| Alto Alegre^a^ | 13 | 149 | 29.7 |
| Amajari^a^ | 13 | 83 | 26.5 |
| Boa Vista^a^ | 13 | 2039 | 21.3 |
| Bonfim^a^ | 13 | 61 | 18.2 |
| Cantá^a^ | 13 | 68 | 15.5 |
| Caracaraí^a^ | 13 | 63 | 11.9 |
| Caroebe^a^ | 13 | 13 | 5.1 |
| Iracema^a^ | 13 | 17 | 6.8 |
| Mucajaí^a^ | 13 | 47 | 10.6 |
| Normandia^a^ | 13 | 44 | 14.2 |
| Pacaraima^a^ | 13 | 113 | 28.5 |
| Rorainópolis^a^ | 13 | 83 | 10.7 |
| São João da Baliza^a^ | 13 | 15 | 7.1 |
| São Luiz^a^ | 13 | 15 | 8.0 |
| Uiramutã^a^ | 13 | 34 | 11.5 |
| Northeast – Bahia (n = 22) | | | |
| Arataca | 7 | 26 | 33.5 |
| Barro Preto | 7 | 16 | 35.1 |
| Buerarema | 7 | 51 | 42.7 |
| Camacan | 7 | 85 | 43.8 |
| Ibicaraí | 7 | 57 | 34.4 |
| Ilhéus | 7 | 670 | 50.8 |
| Itabuna | 7 | 845 | 59.1 |
| Itaju do Colônia | 7 | 17 | 35.1 |
| Itajuípe | 7 | 66 | 46.2 |
| Itaparica | 7 | 67 | 46.1 |
| Itapé | 7 | 32 | 41.8 |
| Jussari | 7 | 13 | 29.5 |
| Lauro de Freitas | 7 | 505 | 38.3 |
| Madre de Deus | 7 | 59 | 46.1 |
| Salvador | 7 | 10,565 | 56.5 |
| Santa Luzia | 7 | 28 | 28.4 |
| Simões Filho | 7 | 357 | 42.2 |
| São José da Vitória | 7 | 13 | 32.1 |
| Una | 7 | 72 | 48.2 |
| Belmonte | 3 | 38 | 59.2 |
| Eunápolis | 3 | 179 | 53.9 |
| Santa Cruz Cabrália | 3 | 28 | 32.9 |
| Northeast – Ceará (n = 9) | | | |
| Aquiraz | 4 | 149 | 47.3 |
| Eusébio | 4 | 69 | 28.4 |
| Fortaleza | 4 | 5460 | 53.6 |
| Guaiúba | 4 | 30 | 30.7 |
| Horizonte | 4 | 73 | 27.6 |
| Itaitinga | 4 | 196 | 98.9 |
| Maracanaú | 4 | 403 | 43.9 |
| Pacajus | 4 | 98 | 35.9 |
| Pacatuba | 4 | 114 | 36.3 |
| Northeast – Maranhão (n = 8) | | | |
| Axixá | 1 | 2 | 16.8 |
| Bacabeira | 1 | 2 | 12.4 |
| Cachoeira Grande | 1 | 1 | 10.8 |
| Presidente Juscelino | 1 | 1 | 8.5 |
| Rosário | 1 | 12 | 29.8 |
| Santa Rita | 1 | 1 | 2.8 |
| São José de Ribamar | 1 | 79 | 39.1 |
| São Luís | 1 | 535 | 50.4 |
| Northeast – Pernambuco (n = 10) | | | |
| Abreu e Lima | 12 | 1018 | 42.4 |
| Camaragibe | 12 | 861 | 23.6 |
| Igarassu | 12 | 557 | 20.7 |
| Ilha Dde Itamaracá | 12 | 1117 | 196.9 |
| Itapissuma | 12 | 979 | 153.6 |
| Jaboatão dos Guararapes | 12 | 4156 | 25.7 |
| Olinda | 12 | 3051 | 33.6 |
| Paulista | 12 | 1729 | 21.7 |
| Recife | 12 | 15,720 | 41.3 |
| São Lourenço da Mata | 12 | 709 | 26.6 |
| Southeast - Rio de Janeiro (n = 15) | | | |
| Belford Roxo^a^ | 13 | 3579 | 27.0 |
| Duque de Caxias^a^ | 13 | 8510 | 36.9 |
| Japeri^a^ | 13 | 1504 | 57.2 |
| Magé^a^ | 13 | 2048 | 32.7 |
| Mesquita^a^ | 13 | 1588 | 34.3 |
| Nilópolis^a^ | 13 | 1302 | 31.0 |
| Nova Iguaçu^a^ | 13 | 7035 | 32.5 |
| Queimados^a^ | 13 | 1031 | 27.1 |
| Rio de Janeiro^a^ | 13 | 68,822 | 39.5 |
| São João de Meriti^a^ | 13 | 4282 | 34.7 |
| Engenheiro Paulo de Frontin | 6 | 43 | 54.8 |
| Itaguaí | 6 | 340 | 47.0 |
| Miguel Pereira | 6 | 20 | 12.6 |
| Paracambi | 6 | 363 | 130.0 |
| Seropédica | 6 | 221 | 46.1 |
| Southeast - São Paulo (n = 85) | | | |
| Carapicuíba^a^ | 13 | 2050 | 20.1 |
| Cotia^a^ | 13 | 760 | 12.1 |
| Cubatão^a^ | 13 | 1180 | 37.8 |
| Diadema^a^ | 13 | 1704 | 16.2 |
| Embu das Artes^a^ | 13 | 1280 | 19.2 |
| Embu-Guaçu^a^ | 13 | 324 | 18.6 |
| Guarujá^a^ | 13 | 3202 | 41.0 |
| Ibiúna^a^ | 13 | 162 | 8.2 |
| Iguape^a^ | 13 | 210 | 26.9 |
| Itanhaém^a^ | 13 | 1063 | 40.0 |
| Itapecerica da Serra^a^ | 13 | 816 | 19.3 |
| Itapevi^a^ | 13 | 1273 | 21.8 |
| Itariri^a^ | 13 | 74 | 17.9 |
| Jandira^a^ | 13 | 384 | 12.6 |
| Juquitiba^a^ | 13 | 98 | 13.0 |
| Juquiá^a^ | 13 | 89 | 18.3 |
| Miracatu^a^ | 13 | 103 | 19.7 |
| Mongaguá^a^ | 13 | 1051 | 73.3 |
| Osasco^a^ | 13 | 3178 | 16.8 |
| Pedro de Toledo^a^ | 13 | 44 | 15.3 |
| Peruíbe^a^ | 13 | 718 | 42.0 |
| Piedade^a^ | 13 | 94 | 6.7 |
| Pilar do Sul^a^ | 13 | 47 | 6.5 |
| Praia Grande^a^ | 13 | 3206 | 39.5 |
| Registro^a^ | 13 | 432 | 28.3 |
| Santo André^a^ | 13 | 2307 | 12.0 |
| Santos^a^ | 13 | 3514 | 31.1 |
| São Bernardo do Campo^a^ | 13 | 2456 | 11.5 |
| São Caetano do Sul^a^ | 13 | 276 | 6.5 |
| São Lourenço da Serra^a^ | 13 | 32 | 8.0 |
| São Paulo^a^ | 13 | 67,403 | 21.8 |
| São Roque^a^ | 13 | 151 | 7.1 |
| São Vicente^a^ | 13 | 5000 | 55.9 |
| Taboão da Serra^a^ | 13 | 1237 | 17.7 |
| Tapiraí^a^ | 13 | 14 | 6.6 |
| Vargem Grande Paulista^a^ | 13 | 111 | 8.9 |
| Balbinos | 11 | 344 | 262.5 |
| Getulina | 11 | 261 | 73.3 |
| Guaimbê | 11 | 16 | 8.7 |
| Júlio Mesquita | 11 | 13 | 8.9 |
| Lavínia | 11 | 638 | 203.7 |
| Mirandópolis | 11 | 555 | 58.5 |
| Pirajuí | 11 | 454 | 59.6 |
| Reginópolis | 11 | 199 | 78.3 |
| Uru | 11 | 1 | 2.3 |
| Valparaíso | 11 | 409 | 51.7 |
| Álvaro de Carvalho | 11 | 217 | 135.6 |
| Guaraçaí | 10 | 9 | 5.5 |
| Irapuru | 10 | 225 | 147.5 |
| Junqueirópolis | 10 | 275 | 68.8 |
| Monte Castelo | 10 | 5 | 6.0 |
| Nova Guataporanga | 10 | 1 | 2.3 |
| Nova Independência | 10 | 22 | 28.6 |
| Pacaembu | 10 | 508 | 177.2 |
| Tupi Paulista | 10 | 298 | 97.1 |
| Adamantina | 7 | 26 | 10.5 |
| Anhumas | 7 | 0 | 0.0 |
| Bento de Abreu | 7 | 3 | 15.9 |
| Dracena | 7 | 217 | 67.9 |
| Emilianópolis | 7 | 5 | 23.1 |
| Estrela do Norte | 7 | 1 | 5.2 |
| Flora Rica | 7 | 0 | 0.0 |
| Flórida Paulista | 7 | 193 | 208.2 |
| Indiana | 7 | 8 | 22.6 |
| Inúbia Paulista | 7 | 2 | 7.7 |
| Lucélia | 7 | 148 | 103.6 |
| Mariápolis | 7 | 3 | 11.3 |
| Martinópolis | 7 | 310 | 176.4 |
| Nantes | 7 | 3 | 15.7 |
| Narandiba | 7 | 8 | 22.7 |
| Ouro Verde | 7 | 8 | 14.4 |
| Pirapozinho | 7 | 30 | 16.7 |
| Pracinha | 7 | 108 | 550.4 |
| Presidente Bernardes | 7 | 293 | 293.0 |
| Regente Feijó | 7 | 23 | 16.7 |
| Ribeirão dos Índios | 7 | 3 | 20.0 |
| Salmourão | 7 | 3 | 8.7 |
| Santo Expedito | 7 | 1 | 4.8 |
| Taciba | 7 | 5 | 11.8 |
| Tarabai | 7 | 10 | 21.3 |
| Álvares Machado | 7 | 18 | 9.9 |
| Andradina | 3 | 57 | 32.1 |
| Castilho | 3 | 9 | 15.5 |
| Murutinga do Sul | 3 | 1 | 8.2 |
| São João do Pau D'alho | 3 | 0 | 0.0 |
| South - Rio Grande do Sul (n = 25) | | | |
| Alvorada | 12 | 1865 | 39.0 |
| Arroio dos Ratos | 12 | 170 | 49.4 |
| Barra do Ribeiro | 12 | 74 | 24.3 |
| Barão do Triunfo | 12 | 8 | 5.0 |
| Cachoeirinha | 12 | 625 | 20.1 |
| Canoas | 12 | 2224 | 26.9 |
| Charqueadas | 12 | 1061 | 121.9 |
| Eldorado do Sul | 12 | 223 | 24.7 |
| Esteio | 12 | 547 | 28.1 |
| Guaíba | 12 | 537 | 23.0 |
| Mariana Pimentel | 12 | 10 | 10.6 |
| Nova Santa Rita | 12 | 110 | 17.6 |
| Porto Alegre | 12 | 13,276 | 38.6 |
| Sapucaia do Sul | 12 | 987 | 30.3 |
| Sentinela do Sul | 12 | 13 | 10.1 |
| Sertão Santana | 12 | 21 | 14.6 |
| São Jerônimo | 12 | 106 | 19.9 |
| Tapes | 12 | 85 | 21.9 |
| Triunfo | 12 | 88 | 13.4 |
| Viamão | 12 | 1541 | 26.5 |
| Arambaré | 7 | 5 | 18.1 |
| Cerro Grande do Sul | 7 | 8 | 11.4 |
| Capela de Santana | 5 | 23 | 41.6 |
| Montenegro | 5 | 237 | 75.0 |
| São Leopoldo | 5 | 538 | 48.2 |
| Central-West - Mato Grosso (n = 9) | | | |
| Apiacás^a^ | 13 | 35 | 15.4 |
| Colniza^a^ | 13 | 34 | 4.9 |
| Acorizal | 7 | 7 | 19.0 |
| Cuiabá | 7 | 2235 | 51.5 |
| Jangada | 7 | 12 | 22.4 |
| Nossa Senhora do Livramento | 7 | 25 | 30.6 |
| Poconé | 7 | 115 | 51.8 |
| Porto Estrela | 7 | 22 | 91.7 |
| Várzea Grande | 7 | 957 | 48.1 |
| Central-West - Mato Grosso do Sul (n = 37) | | | |
| Amambai^a^ | 13 | 420 | 41.9 |
| Aral Moreira^a^ | 13 | 50 | 17.8 |
| Caarapó^a^ | 13 | 176 | 23.1 |
| Coronel Sapucaia^a^ | 13 | 168 | 44.6 |
| Laguna Carapã^a^ | 13 | 22 | 12.4 |
| Iguatemi | 7 | 30 | 29.3 |
| Juti | 7 | 19 | 42.0 |
| Paranhos | 7 | 45 | 49.3 |
| Tacuru | 7 | 69 | 91.1 |
| Anastácio | 6 | 99 | 67.2 |
| Antônio João | 6 | 16 | 29.7 |
| Aquidauana | 6 | 184 | 64.2 |
| Bela Vista | 6 | 33 | 23.9 |
| Bodoquena | 6 | 5 | 9.8 |
| Bonito | 6 | 38 | 28.6 |
| Campo Grande | 6 | 2576 | 48.9 |
| Caracol | 6 | 5 | 15.6 |
| Corguinho | 6 | 4 | 13.5 |
| Corumbá | 6 | 535 | 86.3 |
| Dois Irmãos do Buriti | 6 | 79 | 119.9 |
| Douradina | 6 | 8 | 23.8 |
| Dourados | 6 | 710 | 52.0 |
| Fátima do Sul | 6 | 22 | 18.0 |
| Guia Lopes da Laguna | 6 | 16 | 25.6 |
| Itaporã | 6 | 23 | 16.4 |
| Jardim | 6 | 81 | 54.4 |
| Ladário | 6 | 47 | 37.2 |
| Maracaju | 6 | 53 | 20.8 |
| Miranda | 6 | 76 | 47.8 |
| Nioaque | 6 | 12 | 14.2 |
| Ponta Porã | 6 | 138 | 26.1 |
| Porto Murtinho | 6 | 18 | 20.9 |
| Rio Brilhante | 6 | 56 | 26.5 |
| Rio Negro | 6 | 11 | 36.2 |
| Rochedo | 6 | 12 | 40.1 |
| Sidrolândia | 6 | 64 | 23.1 |
| Terenos | 6 | 26 | 24.3 |

^a^ High-priority municipality (n = 212).

^b^ Incidence rate per 100,000 inhabitants-years.

| **S5 Table.** Spatial variation in temporal trends clusters for the occurrence of pulmonary tuberculosis in Brazil, 2010-2023. | | | | | | | | | | | |
| --- | --- | --- | --- | --- | --- | --- | --- | --- | --- | --- | --- |
| **Cluster type** | **Number of municipalities** | **State cluster (N)** | **Center/radius (km)** | **Observed** | **Expected** | **Rate^a^** | **ITT^b^** | **OTT^b^** | **RR** | **LLR** | **p-value** |
| Most likely | 493 | Acre (14), Amapá (16), Amazonas (62), Maranhão (101), Mato Grosso (41), Pará (144), Rondônia (48), Roraima (15), Tocantins (52) | Manaus/1966.72 | 124712 | 86438.6 | 45.3 | 1.99 | -0.20 | 1.51 | 407.4 | 0.001 |
| 1st secondary | 5 | Rio Grande do Norte (5) | Sen. Georgino Avelino/16.56 | 1032 | 275.3 | 117.8 | 19.35 | 0.10 | 3.75 | 209.6 | 0.001 |
| 2nd secondary | 22 | Mato Grosso do Sul (22) | Rio Negro/201.23 | 7010 | 5772.5 | 38.2 | 6.15 | 0.08 | 1.22 | 189.6 | 0.001 |
| 3th secondary | 32 | Espírito Santo (26), Rio de Janeiro (6) | Marataízes/91.36 | 9249 | 8711.7 | 33.4 | 4.81 | 0.07 | 1.06 | 154.8 | 0.001 |
| 4th secondary | 23 | Sergipe (23) | Santa Rosa de Lima/37.42 | 3226 | 2679.5 | 37.8 | 7.21 | 0.10 | 1.20 | 117.7 | 0.001 |
| 5th secondary | 19 | Rio de Janeiro (16), São Paulo (3) | Maranguatiba /62.38 | 84864 | 37077.5 | 71.9 | 1.24 | 0.04 | 2.42 | 88.1 | 0.001 |
| 6th secondary | 229 | Rio Grande do Sul (211), Santa Catarina (18) | São Jorge/142.3 | 8030 | 13667.1 | 18.5 | 3.75 | 0.09 | 0.58 | 82.2 | 0.001 |
| 7th secondary | 152 | Goiás (9), Minas Gerais (104), São Paulo (39) | Perdizes/209.39 | 7388 | 19407.7 | 12.0 | 3.49 | 0.10 | 0.38 | 65.1 | 0.001 |
| 8th secondary | 112 | Paraíba (47), Pernambuco (65) | Casinhas/89.71 | 17025 | 16142.3 | 33.1 | 2.29 | 0.08 | 1.06 | 64.1 | 0.001 |
| 9th secondary | 96 | Minas Gerais (74), Rio de Janeiro (22) | Bicas/95.6 | 9380 | 12110.5 | 24.3 | 2.94 | 0.09 | 0.77 | 58.5 | 0.001 |
| 10th secondary | 6 | Paraná (5), São Paulo (1) | Leópolis/23.87 | 305 | 348.9 | 27.5 | 17.80 | 0.12 | 0.87 | 57.6 | 0.001 |
| 11th secondary | 13 | Ceará (13) | Palmácia/35.34 | 3134 | 1841.0 | 53.5 | 4.58 | 0.11 | 1.70 | 47.4 | 0.001 |
| 12th secondary | 16 | São Paulo (16) | Mongaguá/46.75 | 100871 | 70619.9 | 44.9 | 0.79 | 0.06 | 1.48 | 38.5 | 0.001 |
| 13th secondary | 125 | Rio Grande do Sul (122), Santa Catarina (3) | Guarruchos  /236.08 | 3566 | 7098.0 | 15.8 | 3.30 | 0.11 | 0.50 | 28.1 | 0.001 |
| 14th secondary | 10 | São Paulo (10) | Itaí/46.7 | 748 | 735.1 | 32.0 | 6.84 | 0.12 | 1.02 | 24.7 | 0.001 |
| 15th secondary | 12 | Rio Grande do Sul (12) | Manquiné/33.1 | 1056 | 839.0 | 39.5 | 5.61 | 0.12 | 1.26 | 23.6 | 0.001 |
| 16th secondary | 96 | Paraná (96) | Cândido Abreu/143.02 | 5721 | 11667.3 | 15.4 | 2.35 | 0.11 | 0.49 | 22.5 | 0.001 |
| 17th secondary | 70 | Goiás (69), Minas Gerais (1) | Santo Antônio da Barra/159.55 | 1835 | 5434.7 | 10.6 | 4.01 | 0.12 | 0.34 | 21.1 | 0.001 |
| 18th secondary | 4 | Minas Gerais (4) | Gov. Valadares/30.62 | 1265 | 1270.5 | 31.3 | 4.76 | 0.12 | 1.00 | 20.7 | 0.001 |
| 19th secondary | 17 | Santa Catarina (17) | José Boiteux/39.68 | 346 | 1130.4 | 9.6 | 7.71 | 0.12 | 0.31 | 14.3 | 0.006 |
| 20th secondary | 13 | São Paulo (13) | Itapeúna/61.18 | 1097 | 1219.4 | 28.3 | 4.15 | 0.12 | 0.90 | 13.6 | 0.009 |
| 21th secondary | 3 | Minas Gerais (3) | Igarapé/11.95 | 291 | 442.1 | 20.7 | 7.77 | 0.12 | 0.66 | 12.0 | 0.043 |

*Abbreviations:* ITT, inside time trend; OTT, outside time trend; km, kilometers; LLR, log-likelihood ratios; RR, relative risk.

^a^ Incidence rate adjusted by sex and age group per 100,000 inhabitants-years; ^b^ % per year
