# Supplementary material for: Space-time clustering and temporal trend analysis of pulmonary tuberculosis in Brazil, 2010–2023
Source: PLoS One. 2026 Jan 8;21(1):e0339784. doi: 10.1371/journal.pone.0339784 (PMC12782390; doi:10.1371/journal.pone.0339784)
Supplement: Attachment — Submitted filename: Response_to_Reviewers.docx [file pone.0339784.s003.docx]

**Plos One**

Title: **Space-time clustering and temporal trend analysis of pulmonary tuberculosis in Brazil, 2010–2023**

PONE-D-25-27079.R1

Point-by-point response

**COMMENTS TO THE AUTHOR:**
Associate Editor: thanks for addressing the comments.

We note that Figures 2, 3, 4 and 6 in your submission contain [map/satellite] images which may be copyrighted. All PLOS content is published under the Creative Commons Attribution License (CC BY 4.0), which means that the manuscript, images, and Supporting Information files will be freely available online, and any third party is permitted to access, download, copy, distribute, and use these materials in any way, even commercially, with proper attribution. For these reasons, we cannot publish previously copyrighted maps or satellite images created using proprietary data, such as Google software (Google Maps, Street View, and Earth). For more information, see our copyright guidelines: http://journals.plos.org/plosone/s/licenses-and-copyright.

**Response:**

We appreciate the reviewer’s concern regarding copyright compliance for the maps.
We clarify that all cartographic bases used in the figures were obtained from the public database of the Brazilian Institute of Geography and Statistics (IBGE), which provides open-access shapefiles under public domain.
The thematic maps included in the manuscript were entirely produced by the authors based on these datasets, without the use of any copyrighted or proprietary map sources such as Google Maps or similar services.
To make this explicit, we have revised the Methods section to include the following sentence (lines 176–180):
“All the cartographic bases used for constructing the thematic maps were obtained from the official and publicly available database of the IBGE (https://www.ibge.gov.br/geociencias/organizacao-do-territorio/malhas-territoriais.html). These shapefiles are open-access and released under public domain. All maps presented in this study were produced by the authors using these public datasets.”

In addition, the figure captions have been updated to include the note:

“Source: Brazilian Institute of Geography and Statistics (IBGE), Malha Municipal (municipal territorial mesh).”

- Is the variable source described by the location of probable infection? Is there a chance that this data is recorded by the municipality of residence?

**Response:**

We appreciate the editor’s thoughtful comment.
The SINAN-TB (Brazilian Notifiable Diseases Information System for tuberculosis) does not include a specific variable indicating the probable location of infection. Consequently, we used the municipality of residence as a proxy for the probable place of infection.
This methodological choice was based on both the epidemiological characteristics of tuberculosis and the structure of the available surveillance data. Tuberculosis is a chronic infectious disease with prolonged exposure periods, and transmission often occurs in the patient’s usual living or working environment, which typically coincides with their place of residence.
Using the municipality of residence allows for consistent spatial aggregation and comparison across cases, enabling the identification of geographic patterns of disease occurrence and potential clusters of transmission.
Nevertheless, we acknowledge that in some instances the municipality of infection may differ from the municipality of residence, particularly in highly mobile populations or cases involving intermunicipal transmission, which represents a limitation inherent to the available data. We hope this explanation adequately addresses the editor’s concern.

- Although the Discussion briefly mentions the implications of prisons, could you elaborate on how this relates to your results? From the map, there appear to be hotspots in areas with a high concentration of prisons. Please consider adding the locations of prisons in Brazil and examining whether these areas show stronger associations in your analyses. Alternatively, if such an analysis is beyond scope, could you at least cite published studies that document similar patterns?

**Response:**

We thank the editor for the thoughtful comment and for highlighting the relevance of prisons as critical environments for tuberculosis transmission in Brazil. We fully agree that incarceration is an important social determinant of TB and recognize the existence of geographical overlap between high TB burden areas and the location of prison facilities. However, a detailed investigation of this relationship goes beyond the scope of the present study.

The SINAN-TB database does not include a specific field identifying people deprived of liberty (POP_LIBER) before 2015, and even in more recent years, the completeness of this information remains heterogeneous across municipalities. Incorporating this variable would thus restrict the study period and reduce data comparability. Moreover, our analysis included all age groups, while the incarcerated population in Brazil is legally restricted to individuals aged 18 years and older. Restricting the dataset to adults would substantially alter our inclusion criteria and analytical design. Additionally, an appropriate assessment of incarceration effects would require adjustment for additional covariates; such as socioeconomic vulnerability, population density, and incarceration rates by municipality; that are not consistently available for all years analyzed. Socioeconomic and demographic indicators are derived from the 2010 national census, and although a new census was conducted in 2022, its detailed datasets have not yet been released. Consequently, incorporating these variables would restrict the temporal scope of the study and compromise comparability across the analyzed period.

For these reasons, we maintained our focus on the spatial and temporal distribution of pulmonary TB in the general population, while acknowledging that prisons represent potential amplifiers of TB transmission in certain regions. We appreciate the editor’s valuable suggestions, which raise important hypotheses that can be addressed in future studies specifically designed to integrate incarceration data and associated socioeconomic factors.

In line with the editor’s recommendation, we have expanded the Discussion to include references to studies that have documented the strong association between incarceration and TB in Brazil. Please see lines 407­–413.

“Although this study did not directly assess the role of prisons in TB transmission, it is essential to acknowledge that incarceration remains a major structural driver of TB in Brazil [36]. Evidence demonstrated that incarcerated individuals experience extremely high TB incidence, that transmission networks link prisons and surrounding municipalities, and that these patterns are influenced by social inequality and mobility of the incarcerated population [8,37]. Future studies could build upon our findings by incorporating incarceration-related indicators to further clarify the spatial interplay between institutional and community transmission.”

- Rename the figure names as they appear in the manuscript;

- For the figure 'SVTT_2010a2023', consider including the capitals of each state or at least a point that represent them. In the legend, remove the border of polygons and add some space between them, as in the map, they don't have a border.  

- Change '—' for '–'

**Response:**

We thank the editor for the suggestions. All requested changes have been implemented. In Figures 4 and Figure 6, the capitals of each state were added (represented by points), and the polygon borders in the legend were removed, with additional spacing introduced between items to ensure consistency with the map’s visual layout. Additionally, all em dashes (“—”) have been replaced with en dashes (“–”) throughout the manuscript.

**COMMENTS TO THE AUTHOR:**
Reviewer #1: thanks for addressing the comments.

1. Abstract

I recommend avoiding the use of abbreviations in the abstract. The journal advises minimizing abbreviations whenever possible.

I suggest making the conclusion more concise and highlighting the practical implications of the findings, such as the prioritization of specific areas for intervention.

**Response:**

We thank the reviewer for this thoughtful suggestion regarding the abbreviations used in the Abstract. We carefully reviewed this section and maintained the three abbreviations to ensure clarity and fluency, as they are widely recognized and frequently used in the tuberculosis literature.

The abbreviation SINAN (Brazil’s Notifiable Diseases Information System) was kept only once to indicate the original Portuguese name of the database and is not repeated elsewhere in the manuscript. The abbreviation PTB (pulmonary tuberculosis) was maintained to improve readability, as the full term is lengthy and recurrent throughout the text, which could make the reading cumbersome. The abbreviation RR (relative risk) appears only once after its first mention, serving mainly to facilitate comprehension without redundancy. Additionally, the Conclusion has been revised to make it more concise and to emphasize the practical implications of our findings, particularly the identification of priority areas for targeted surveillance and intervention, as shown in the updated version above (lines 20­­–23, main manuscript):

“The study identified persistent and expanding patterns of PTB in Brazil, revealing specific areas with higher burden and increasing trends that should be prioritized. These findings reinforce the need for regionally tailored surveillance and control strategies to ensure a more effective response to tuberculosis.”

2. Introduction

I recommend strengthening the theoretical background regarding the social and structural inequalities that influence the distribution of TB in Brazil, particularly in the territories identified as priorities.

**Response:**

We thank the reviewer for this valuable comment and fully agree that social and structural inequalities play a central role in shaping the distribution of tuberculosis in Brazil. In response, we have strengthened the theoretical background in the Introduction to better contextualize how socioeconomic disparities, housing conditions, access to healthcare, incarceration, and regional inequalities influence TB burden and persistence across Brazilian territories.

Specifically, we added a paragraph highlighting the interplay between poverty, overcrowding, urban vulnerability, and limitations of health and social systems, as well as their contribution to sustaining endemic transmission in priority areas such as metropolitan regions and the Legal Amazon. These revisions aim to provide a clearer conceptual link between the social determinants of health and the spatial heterogeneity of TB identified in our analyses.

The revised Introduction now reads (excerpt), lines 34–46:

“…

In a country as large and diverse as Brazil, TB distribution is highly heterogeneous, reflecting deep social and structural inequalities. The persistence of poverty, housing shortages, overcrowded environments, malnutrition, and limited access to health services contributes to maintaining endemic transmission, particularly in urban peripheries and territories with poor living conditions [5–7]. Incarceration, migration, and informal labor further exacerbate vulnerability, while regional disparities in health-system capacity and social protection reinforce unequal exposure and outcomes [8,9]. These inequalities explain why certain municipalities and regions, such as large metropolitan areas, border zones, and parts of the Legal Amazon, have remained persistent TB hotspots over time [5,6,10]. Understanding these spatial disparities is therefore crucial for advancing toward equitable TB control and aligning public health strategies with the broader social determinants of health.

…”

We believe these additions strengthen the theoretical grounding of the study and align the Introduction more closely with the reviewer’s recommendation.

3. Materials and Methods

I suggest reorganizing this section into clearer subsections to facilitate readability (e.g., “Unit of analysis,” “Data sources,” “Statistical analysis,” “Spatial analysis”).

**Response:**

We thank the reviewer for the thoughtful suggestion regarding the organization of the Materials and Methods section. The manuscript is already structured into specific subsections that correspond closely to the topics mentioned, ensuring clarity and logical flow.

The subsection “Study design and setting” encompasses the unit of analysis, providing a broader contextualization that includes the type of study, the analytical unit (municipalities), and the geographic and epidemiological setting in which the analysis was conducted. Restricting this subsection to the unit of analysis alone would narrow its scope and fragment information that is conceptually interdependent.

The subsection “Study population and data sources” addresses the sources of information used, including surveillance data (SINAN-TB) and demographic indicators, while the “Variables” subsection was intentionally kept separate to describe in detail the indicators derived and their analytical relevance, information necessary for understanding the statistical and spatial modeling procedures.

Finally, the “Statistical analysis” section already includes the recommended analytical components, organized into the following subsections for clarity:

- Time series analysis (to assess temporal trends using segmented regression and seasonal patterns),
- Spatial autocorrelation analysis (using global and local Moran’s I to identify spatial dependence and clustering), and
- Space-time scan statistic analysis (to detect clusters of high or low risk based on Poisson models).

We believe that the current structure provides an integrated and accessible presentation of the methods, allowing readers to follow the analytical process step by step while preserving conceptual coherence.

I suggest justifying and referencing the exclusion of clusters with ≤2 municipalities, as the rationale presented (random fluctuation) lacks an explicit methodological foundation.

**Response:**

Thank you for your comment. We recognize the importance of providing a stronger methodological foundation for the exclusion of clusters composed of two or fewer municipalities.

In response, we have expanded the rationale in the Methods section to reflect both methodological guidance and established practice. Although our original justification referenced the potential for random fluctuation, this decision is also grounded in recommendations from the official SaTScan user manual (page 18), which advises reporting only clusters with at least two locations and a minimum number of cases, and with p < 0.05 (https://www.satscan.org/techdoc.html). Therefore, this approach aligns with the software’s default parameters and aims to enhance the statistical robustness and epidemiological interpretability of the detected clusters.

Moreover, clusters formed by only one or two spatial units are widely considered less reliable due to their susceptibility to random variability and the modifiable areal unit problem (MAUP). These clusters are more prone to unstable incidence rates and may lead to spurious results. Similar approaches are documented in the literature, for instance, Huang et al. (2009 [doi:10.1198/jasa.2009.ap07613]) explicitly defined a minimum cluster size of two major statistical areas (MSSAs) or 10 subdivisions to avoid overinterpreting potentially spurious clusters of minimal spatial extent. Likewise, Paiva et al. (2022 [doi:10.1371/journal.pone.0247894]), in their spatiotemporal analysis of tuberculosis in Brazil, excluded clusters with one or two municipalities to mitigate statistical instability and improve the interpretability of the results.

Thus, by applying this exclusion criterion, we follow standard methodological practice to reduce statistical noise and prioritize more stable spatial patterns. The manuscript has been updated to reflect this rationale and now includes appropriate references to support this decision (lines 166­–170).

“To mitigate statistical noise and enhance the interpretability of results, clusters composed of two or fewer municipalities were excluded from the analysis, as recommended [22]. Very small clusters are more susceptible to random variability and to the modifiable areal unit problem (MAUP), which can lead to unstable incidence estimates and potentially spurious patterns or false positives arising from random fluctuation [5,25].”

I recommend detailing the procedure used for the proportional redistribution of missing data (sex, age, municipality), including the statistical basis and potential limitations of this method.

**Response:**

Thank you for this helpful suggestion. We have expanded the Methods section to describe in detail the proportional redistribution procedure applied to records with missing sex, age, or municipality of residence, including its statistical assumptions and limitations.

Briefly, for each analytical stratum, the small number of missing values was redistributed in proportion to the observed distribution within the same group: (i) for sex and age, within the same municipality and calendar year; and (ii) for municipality of residence, within the same state and calendar year. This approach is equivalent to single imputation using empirical (observed) category frequencies under a Missing Completely at Random (MCAR) or Missing at Random (MAR) framework. It preserves totals and prevents artificial deflation of incidence rates in strata with minimal missingness.

We also clarify that proportional redistribution does not incorporate imputation uncertainty and could be biased if missingness were Missing Not at Random (MNAR). However, given the extremely low proportion of missing data (sex: 0.008%; age: 0.03%; residence: 0.02%), the uniformity of missingness patterns across strata, and the structured data collection workflow, we find no evidence of an MNAR mechanism.

The following text has been added to the manuscript (lines 82–91), with references to support the methodological approach:

“…

Records with missing sex (n=76; 0.008%), age (n=235; 0.03%), or municipality of residence (n=168; 0.02%) were handled by proportional redistribution: (i) for sex and age, within the same municipality and calendar year; and (ii) for municipality of residence, within the same state and calendar year. This is equivalent to single imputation using empirical frequencies under missing completely at random (MCAR) or missing at random (MAR) assumptions, preserving totals and avoiding artificial rate deflation [16]. Two caveats apply: it does not propagate imputation uncertainty and could be biased if missingness were missing not at random (MNAR) [17]. However, given the extremely low missingness, uniform patterns across strata, and the data-collection workflow, we find no evidence of an MNAR mechanism [18].”

I suggest including the limitations of the space-time scan statistic (STSS), particularly regarding its sensitivity to the spatial window boundaries and the lack of contextual variables.

**Response:**

Thank you for this valuable comment. We agree that the space–time scan statistic (STSS) has methodological limitations that should be acknowledged. Specifically, its results can be sensitive to the definition of spatial and temporal scanning windows, which may influence the size and delineation of detected clusters. To mitigate this effect, we followed SaTScan’s recommended approach of using relative spatial and temporal window limits based on the population at risk and the study period, thereby enhancing robustness and comparability across regions.

Moreover, while the STSS is well suited for identifying statistically significant clusters, it does not incorporate contextual or structural determinants that may underlie the observed patterns—such as internal migration flows, population mobility, barriers to health-care access, and socioeconomic disparities. To partially address this limitation, we included sex and age as covariates in the analysis, controlling for key demographic differences that might otherwise confound spatial patterns.

Accordingly, the Strengths and Limitations section was revised as follows (lines 479–486):

“Third, the space–time scan statistic is sensitive to the definition of spatial and temporal scanning windows, which may influence cluster size and boundaries [25]. Furthermore, although sex and age were included as covariates, the method cannot fully capture relevant contextual determinants, such as internal migration flows, population mobility, barriers to health-care access, and local environmental or structural factors, that also influence disease dynamics. Future studies should integrate such contextual variables to provide a more comprehensive, place-based understanding of TB persistence across territories.”

We believe this revision provides a clearer discussion of the limitations and methodological considerations of the STSS, and we hope these changes address the reviewer’s concern.

4. Results

I recommend including a quantitative synthesis of the national-level results, such as the overall mean and annual trend of TB incidence in Brazil.

**Response:**

Thank you for this constructive suggestion. We agree that summarizing the national-level results strengthens the clarity of the Results section.

The overall mean incidence rate was already presented in the manuscript under the subsection Temporal patterns and trend (line 209, Table 1). To address the reviewer’s comment and enhance the quantitative synthesis, we have now also included the Average Annual Percent Change (AAPC) to describe the national temporal trend.

The revised text reads as follows (lines 209–211):

“The mean PTB incidence rate during the study period was 30.3 cases per 100,000 inhabitants-years (ranging from 31.2 in 2010 to 33.6 per 100,000 in 2023). Overall, the long-term trend was stable (AAPC = 0.87; 95% CI: –0.04 to 1.34).”

- I suggest condensing the discussion section to avoid repetition of the results and to make the text more analytical and less descriptive.

**Response:**

Thank you for this valuable suggestion. Following your recommendation, we carefully revised and condensed the Discussion section to reduce redundancy and strengthen its analytical focus. Descriptive passages that repeated results were streamlined, and transitions were refined to improve clarity and flow.

Additionally, as suggested by the Academic Editor, we incorporated a new paragraph discussing the role of prisons in tuberculosis transmission, providing contextual support from the literature and linking it to our findings in high-incidence areas.

We believe these changes make the Discussion more concise, interpretive, and aligned with the journal’s standards, and we hope these revisions address the reviewer’s concern.

- I recommend explicitly addressing the methodological limitations of the study, including ecological bias and the modifiable areal unit problem (MAUP).

**Response:**

Thank you for this important comment. The manuscript already addresses this issue in the Strengths and Limitations section, where we discuss the modifiable areal unit problem (MAUP). This discussion highlights that spatial results may be influenced by the level of geographic aggregation (e.g., municipal boundaries), which can affect comparability across studies and the interpretation of cluster patterns. However, we have expanded this section to explicitly include ecological bias, clarifying that, as an ecological study, the analyses are based on aggregated data and therefore do not allow for causal inferences at the individual level.

The revised text reads (lines 487–491):

“Fourth, as an ecological study, the analyses are subject to ecological bias, as associations observed at the municipal level may not reflect individual-level relationships. We also acknowledge the modifiable areal unit problem, whereby results can vary depending on the spatial scale or zoning scheme used, potentially influencing the comparability and interpretation of spatial patterns.”

I suggest expanding the discussion on the international and structural implications of the findings, including comparisons with other high TB burden countries in Latin America.

**Response:**

Thank you for this insightful comment. Our revised Discussion section situates Brazil’s TB scenario within the broader regional context of high-burden countries in Latin America. The text now highlights that structural and social inequities, such as poverty, overcrowding, incarceration, migration, and barriers to healthcare, are shared determinants of TB transmission across the region. Recent comparative evidence from Latin American settings shows that countries like Peru and Venezuela face similar challenges in TB control due to concentrated urban poverty, limited access to diagnostic tools, and the impact of social vulnerability on delayed case detection and treatment adherence.

The revised paragraph also discusses the structural implications of our findings, emphasizing that the persistence of spatial clusters in Brazil reflects the same underlying socioeconomic drivers that hinder progress toward WHO’s End TB goals in neighboring countries. Strengthening social protection policies, expanding access to rapid diagnostics, and integrating TB control with broader health and social programs are highlighted as essential strategies that can be informed by Brazil’s spatial evidence.

These points were incorporated into the Discussion section to improve the international comparability and structural interpretation of our findings. The revised section now includes the following text (lines 414–420):

“The spatial and temporal heterogeneity observed in Brazil parallels patterns reported in other high TB burden countries in Latin America, such as Peru, and Venezuela where socioeconomic vulnerability, overcrowding, and fragmented health systems similarly sustain persistent transmission [1,47]. These findings reinforce that structural inequities, including poverty, housing precarity, incarceration, and migration, are regional determinants of TB, and that spatially targeted, intersectoral interventions are crucial for accelerating progress toward the End TB targets across Latin America [34,48].”

We believe this addition strengthens the discussion by explicitly connecting our findings to the regional context and highlighting their broader public health implications.

-I recommend discussing the limitations of the temporal analysis in the context of the COVID-19 pandemic, considering the possible influence of changes in surveillance and reporting systems during that period.

**Response:**

We thank the reviewer for this thoughtful and important comment. The potential influence of the COVID-19 pandemic on TB surveillance, notification, and reporting has already been addressed in the current version of the manuscript, in two distinct sections.

In the Discussion (lines 338–350):

“A downturn in case notifications began in the last quarter of 2019 and deepened in March 2020 with the onset of the COVID-19 pandemic. Public fear of visiting health facilities, social-distancing measures, and the diversion of resources to the COVID-19 response markedly reduced the demand for diagnosis and treatment of other diseases, including TB [30]. Partial suspension of TB surveillance and control activities, interruption of screening campaigns, and limited availability of laboratory tests further contributed to underreporting and a temporary weakening of the TB response, explaining the abrupt decline in notifications during 2020 [31].

Consequently, the PTB incidence rate, previously high but relatively stable, fell sharply between 2020 and 2021, returning to pre-pandemic levels only in 2022. Despite this rebound, the results indicate that, although progress has been made in prevention, diagnosis, and treatment, the decline in TB incidence remains insufficient for Brazil to meet the targets set by the World Health Organization (WHO) and by the National Plan to End TB as a Public Health Problem by 2030 [1,32].”

This section directly discusses the pandemic’s impact on TB surveillance, case detection, and reporting capacity, aligning closely with the reviewer’s suggestion.

In the “Limitations and Strengths” section (lines 491–494):

“In addition, changes in surveillance sensitivity over time may have influenced some of the temporal trends we observed; we could not clearly disentangle the extent to which those trends reflect true changes in disease risk versus fluctuations in case-detection capacity.”

Together, these excerpts explicitly acknowledge the disruptions in surveillance and reporting systems during the COVID-19 period and their possible influence on temporal trends.

Additionally, as suggested by Reviewer 2, we expanded the discussion to include broader structural and policy-related factors that may have shaped TB surveillance and control during the study period. Specifically, we incorporated a new paragraph (lines 377–389) discussing the effects of fiscal austerity policies and the institutional dismantling of the Unified Health System (SUS) under the Bolsonaro administration:

“Complementarily, the increasing trends observed in certain states and regions after 2014 may be associated with fiscal austerity policies that gradually weakened public health financing and reduced the responsiveness of the SUS [41]. As noted, these vulnerabilities were further aggravated during the COVID-19 pandemic. Moreover, political instability and the dismantling of health governance structures in recent years—characterized by successive changes in the Ministry of Health leadership and the circulation of anti-scientific rhetoric—further undermined the capacity of the system to meet population needs. This context had a particularly strong impact in historically vulnerable regions such as the Amazon, where health-system collapse became an imminent risk. Recent evidence supports this interpretation, demonstrating that political denialism contributed to increased mortality, declining vaccination coverage, and the weakening of Brazil’s National Immunization Program [42], factors that can also directly affect TB control by undermining essential surveillance, prevention, and continuity-of-care efforts.”

These additions provide a broader contextual understanding of how macro-level policy decisions and governance crises intersected with the COVID-19 pandemic to influence TB surveillance and reporting capacity in Brazil, and we hope this fully addresses the reviewer’s concern.

6. Conclusion

I suggest rewriting the conclusion to avoid repeating content from the abstract and discussion, and to clearly state the original scientific contribution of the study.

**Response:**

Thank you for this valuable suggestion. We have rewritten the Conclusion to reduce redundancy with the Abstract and Discussion and to explicitly highlight the original contribution of our study. The revised version emphasizes the integration of temporal and spatiotemporal analyses at the municipal level, the identification of persistent high-risk areas across Brazil, and their direct implications for TB surveillance and territorial health planning.

The updated text (lines 452–538) now reads:

“

Conclusion

This study provides the most comprehensive spatiotemporal assessment of PTB in Brazil to date, covering municipal-level data from 2010 to 2023. By integrating time-series, spatial autocorrelation, and space–time scan analyses, it identified persistent and expanding high-risk clusters, particularly in RJMR, in the Legal Amazon, and major urban centers.

The study’s main contribution lies in revealing how these long-term spatial–temporal dynamics expose structural vulnerabilities that sustain TB transmission, offering actionable evidence for territorial health planning. The identification of 212 municipalities as high-priority for public-health interventions enables more precise prioritization of surveillance, diagnostic expansion, and resource allocation within the national TB control strategy.

These findings underscore the need for structured, regionalized, and intersectoral responses focused on strengthening primary care, decentralizing diagnosis, providing timely and adequate treatment, and reducing social inequalities. Importantly, they reaffirm that TB is not only a biomedical issue but also a social and territorial process shaped by the organization of space and the unequal conditions under which people live. Therefore, without sustained, coordinated efforts, Brazil cannot realistically achieve the WHO’s target of eliminating TB as a public-health problem by 2030.

…”

We believe this revision addresses the reviewer’s concern by clearly articulating the novelty and public-health relevance of our findings.

7. References

I suggest reviewing the formatting of the references to ensure full compliance with the style required by PLOS ONE, including the presence of DOIs and other mandatory elements.

**Response:**

Thank you for this observation. We carefully reviewed the entire reference list to ensure full compliance with the PLOS ONE formatting requirements, including the inclusion of DOIs and all mandatory elements. References were managed and formatted using Zotero with the official PLOS ONE citation style plugin, which standardizes references according to the journal’s guidelines.

All entries have been verified for completeness, consistency, and DOI availability.

**COMMENTS TO THE AUTHOR:**
Reviewer #2: thanks for addressing the comments.

-The paper's introduction presents a clear exposition of the problem, citing information at a global level and the scale of analysis (Brazil). It identifies the need for a temporal and spatio-temporal study of PTB and monitoring and control interventions and actions—especially with a focus on regionalized actions, due to Brazil's regional diversity.

**Response:**

We appreciate the reviewer’s positive assessment of the Introduction. In addition, following the suggestions of Reviewer 1, we have strengthened the theoretical background regarding the social and structural inequalities that influence the distribution of tuberculosis in Brazil, particularly in the territories identified as priorities. These revisions aim to provide a deeper contextual basis for the spatial heterogeneity observed in the country. Please see the revised version in the Introduction section.

- The methodology is precise and robust, featuring sophisticated statistical analysis that is recommended for temporal and spatio-temporal analysis studies. The use of secondary data from DATASUS and IBGE allows for a comprehensive analysis of the scale of the study presented. Furthermore, the use of these open-access databases is a strong point of the study, although the authors mention it as a limitation because they do not analyze disaggregated data. In fact, disaggregated data can provide other clues and suggestions for the problem of PTB in Brazil, however, the greater the scale of detail, the lower the level of generalization for creating transmission models and identifying priority areas.

**Response:**

We sincerely thank the reviewer for the positive evaluation of our methodological approach. We appreciate the recognition of the robustness of the temporal and spatiotemporal analyses. We also agree with the reviewer’s insightful comment regarding the trade-off between data disaggregation and generalizability. In response, we have refined the Strengths and Limitations section to clarify that while the use of aggregated data limits fine-grained analyses of local transmission dynamics, it enables a nationwide assessment that is essential for identifying large-scale patterns and priority areas for intervention (lines 475–479):

“…Second, because more disaggregated data, such as residential addresses, were unavailable, all analyses were conducted at the municipal level, which may limit the precise identification of local transmission foci. However, this level of aggregation allows for a broader, more comprehensive assessment, supporting the identification of priority territories for intervention and reducing the loss of generalizability that can occur with highly disaggregated data.

…”

These additions better contextualize the analytical scope of our study and highlight the balance between analytical depth and spatial representativeness.

- The use of Joinpoint Regression, Moran's I, Getis-Ord Gi* and SaTScan tools provides a comprehensive and methodologically powerful analysis. The integration between statistical information and spatial statistics takes into account a key element not mentioned in the work: the geography of PTB. Health-disease processes occur in geographical space and are therefore also conditioned/determined by the form of social production of this space. Literature on the subject of the Health Geography—much enriched in Brazil—may provide clues to this point that I would like to draw attention to.

**Response:**

We sincerely thank the reviewer for this insightful comment. We fully agree that the health–disease process is intrinsically linked to geographic space and influenced by the social production of that space, a central concept in Health Geography that has been particularly well developed in Brazil.

In line with this perspective, we have reinforced this understanding in the Conclusion, emphasizing that TB control requires not only biomedical and surveillance actions but also the consideration of the social and territorial dimensions that shape disease persistence. This addition highlights that spatial analyses, such as those employed in our study, must be interpreted within the broader context of the geography of health and the social organization of space. Please see the updated text in the Conclusion section (lines 532–538):

“…These findings underscore the need for structured, regionalized, and intersectoral responses focused on strengthening primary care, decentralizing diagnosis, providing timely and adequate treatment, and reducing social inequalities. Importantly, they reaffirm that TB is not only a biomedical issue but also a social and territorial process shaped by the organization of space and the unequal conditions under which people live. Therefore, without sustained, coordinated efforts, Brazil cannot realistically achieve the WHO’s target of eliminating TB as a public-health problem by 2030.”

- It is clear that the authors have an understanding of the subjects covered in the paper, and some methodological choices have been prioritized in this extensive and rigorous work. Some of the shortcomings are listed in the limitations section of the paper, such as: i) under-reporting; ii) inconsistency in the data; iii) use of aggregated data; iv) those relating to technique (spatio-temporal analysis techniques, MAUP, etc.).

**Response:**

We sincerely thank the reviewer for the positive and encouraging evaluation of our work. We appreciate the recognition of the methodological rigor and the careful prioritization of analytical choices throughout the study.

In response to the reviewer’s comment, we have further refined the Limitations and Strengths section by incorporating additional points suggested by Reviewer 1. These updates include a clearer discussion of the potential effects of ecological bias, the sensitivity of the space–time scan statistic to parameter selection, and the influence of contextual and structural determinants on disease distribution. Please see the updated Limitations and Strengths section in the revised manuscript.

-Thus, I highlight some limitations/suggestions for the work. In particular, the areas with the highest demographic density, such as the aforementioned favelas in Rio de Janeiro, are proxies for analyzing the incidence of PTB in Brazil - in contrast to the quality of the data produced in remote/rural areas. From this question, which is deeply energized in urbanization debates in urban geography, a question arises: isn't it important to analyze the consistency and completeness of the data? Another question that I think is relevant to think about is whether there are relationships between the urban hierarchies of Brazilian municipalities and the regions of influence of the cities with the incidence of PTB over the time series. Graphs relating these hierarchies to incidence over time could add to a rich debate about how we are constructing Brazilian cities and regions and the impact this has on Brazilian public health—directly affecting the Unified Health System (SUS). Maps relating to this issue of centrality and the search for health services could add to the debate which, despite the exhaustive analysis presented, could be complemented with some social, economic and structural characteristics.

**Response:**

We sincerely thank the reviewer for this insightful and thought-provoking comment. We fully agree that the completeness and consistency of the data are crucial for ensuring the robustness of spatial and spatiotemporal analyses.

In our study, the proportion of incomplete records was extremely low, as detailed in the Variables section and Table S1, which strengthens the reliability of the results. Moreover, we have updated this section to provide a clearer description of how missing data were addressed. Specifically (lines 82–91):

“Records with missing sex (n=76; 0.008%), age (n=235; 0.03%), or municipality of residence (n=168; 0.02%) were handled by proportional redistribution: (i) for sex and age, within the same municipality and calendar year; and (ii) for municipality of residence, within the same state and calendar year. This is equivalent to single imputation using empirical frequencies under missing completely at random (MCAR)/missing at random (MAR) assumptions, preserving totals and avoiding artificial rate deflation [16]. Two caveats apply: it does not propagate imputation uncertainty and could be biased if missingness were missing not at random (MNAR) [17]. However, given the extremely low missingness, uniform patterns across strata, and the data-collection workflow, we find no evidence of an MNAR mechanism [18].”

The use of updated and comprehensive national databases (DATASUS and IBGE) also contributes to improving the completeness and comparability of data across municipalities, reinforcing the reliability of the spatiotemporal assessment.

We recognize that differences in data quality and reporting may exist between highly urbanized areas (e.g., metropolitan favelas) and remote or rural regions. Nevertheless, the minimal missingness observed supports the consistency of the spatial and temporal patterns identified. We also agree that using even more recent data in future analyses will further enhance the reliability of TB surveillance and strengthen the evaluation of underreporting in vulnerable territories.

We further appreciate the reviewer’s valuable reflection on the role of urban hierarchies and regional influence in shaping PTB distribution. Indeed, our findings indicate that the main high-risk clusters are concentrated in Brazil’s metropolitan regions, suggesting that processes of urban concentration and spatial inequality play a central role in sustaining TB transmission. This observation inspired us to develop a complementary study currently under review, focusing specifically on current and projected TB trends up to 2035 in metropolitan and non-metropolitan areas of Brazil, explicitly incorporating urban hierarchy as a key analytical dimension.

We agree that graphs relating these hierarchies to incidence trends could enrich the discussion on how Brazilian cities and regions are being produced and how this spatial organization affects public health, particularly the Unified Health System (SUS). Likewise, maps exploring centrality and health-service access, combined with socioeconomic and structural indicators, could provide an even more comprehensive territorial interpretation. However, these analyses extend beyond the scope and objectives of the present study. Still, we greatly value this perspective, which will be further explored in our forthcoming research.

We believe these clarifications and future directions address the reviewer’s suggestions and further strengthen the methodological transparency and conceptual depth of the manuscript.

- Other types of maps could correlate areas of socio-economic vulnerability with those with a higher incidence of PTB. The relationship in the Amazon region and the Central-West with precarious and slave labor in illegal mining, deforestation, and wildfires could provide clues to social aspects related to a comprehensive analysis of PTB in Brazil. On this subject, we should also pay attention to recent climatic events resulting from the global climate crisis.

**Response:**

We sincerely thank the reviewer for this insightful comment. Although the primary objective of our study was to analyze the spatiotemporal dynamics of pulmonary tuberculosis in Brazil, we recognize that the disease is deeply intertwined with broader social, economic, and environmental determinants. In the revised Discussion section, we expanded our interpretation to better contextualize these structural factors, particularly in the Amazon and Central-West regions, where TB transmission interacts with persistent socioeconomic vulnerabilities, territorial insecurity, and environmental degradation.

Specifically, we incorporated discussion points highlighting how informal and unsafe economic activities—such as illegal mining (*garimpos*), deforestation-related labor, and seasonal agricultural work—are often associated with precarious or forced labor conditions, population mobility, and exposure to environmental pollutants (e.g., silica dust and forest-fire smoke). These factors exacerbate vulnerability to TB through both social and biological pathways, illustrating how precarious work and environmental change jointly sustain disease persistence in frontier regions.

We also cited the recent analytical framework proposed by The Lancet in “Climate change and tuberculosis: an analytical framework” (2025), which emphasizes the links between climate variability, social disruption, and TB risk. This perspective complements our findings by framing TB not only as an infectious disease but also as a manifestation of structural and environmental inequities.

While our analytical scope did not allow for a direct evaluation of these socioenvironmental mechanisms, we believe the expanded discussion provides a more comprehensive and contextual understanding of PTB in Brazil’s high-risk territories. Future studies, including those currently underway by our group, aim to explore the relationships between urban hierarchies, labor precarity, and environmental pressures to further elucidate the territorial determinants of TB transmission.

Specifically, the following passages were inserted into the Discussion (lines 421–434):

“...

Municipalities in the Legal Amazon likewise warrant priority attention in TB control policies, particularly due to persistent adverse socioeconomic indicators that favor transmission [37]. The situation is even more complex in Indigenous communities, where vulnerability stems not only from poverty but also from broader structural factors such as limited land access, territorial insecurity, and climate-change effects [46,47]. These structural vulnerabilities may also interact with environmental stressors, including the effects of climate variability, which have recently been recognized as emerging determinants of TB dynamics [48].

In addition, informal and unsafe economic activities, such as illegal mining (*garimpos*), deforestation-related labor, and seasonal agricultural work, remain widespread in parts of the Amazon and Central-West regions [49]. These contexts often involve precarious or forced labor conditions, population mobility, and intense exposure to silica dust and smoke from forest fires, all of which may amplify TB vulnerability through both social and biological pathways [50]. Addressing these socioenvironmental determinants is therefore essential for developing integrated TB control strategies adapted to these frontier territories.

…”

- With regard to these more political and social issues (although it is well known that health is a social product), in Figure 2 I identified the municipality of Campinápolis (MT) as an area with a high incidence of tuberculosis in all the periods analyzed. In Figure 3, this region of Campinápolis, in the Serra do Roncador, is home to a lot of illegal deforestation, wildfires and indigenous violence against the Xavantes ethnic group. Are these aspects related? Clearly, the aim of this paper was not to problematize this exact issue, but I recommend that you take a closer look at it in a future article and suggest the concept of necropolitics if you wish to delve deeper into these structural aspects.

**Response:**

We thank the reviewer for this insightful and thought-provoking comment. We fully recognize the importance of the socio-political and territorial dimensions highlighted, where high PTB incidence coincides with illegal deforestation, land conflicts, and violence against Indigenous communities.

As noted in the previous response, we have already incorporated a paragraph addressing how socioenvironmental vulnerabilities—such as informal and unsafe economic activities, deforestation-related labor, and exposure to environmental stressors—interact with TB dynamics, especially in the Amazon and Central-West regions. This addition acknowledges the broader structural and environmental context in which TB transmission occurs.

We also agree that a deeper theoretical exploration of these dynamics, including the lens of necropolitics and the role of structural violence, could offer valuable insights into how historical and political processes shape health inequities in Brazil. These critical dimensions, however, go beyond the epidemiological scope of the present study and will be examined in future publications from our research group.

- Another aspect that I think is important to articulate is the analysis of data based on health regions. The authors clearly know and understand that the SUS is a hierarchical, regionalized, and municipalized health system. As such, resources for surveillance, education, and monitoring of PTB (and other diseases) go through these specific regional departments. If the data were also articulated regionally, it could contribute to the final recommendations made in the paper.

**Response:**

We thank the reviewer for this valuable suggestion and fully acknowledge the importance of regionalized health governance in Brazil’s Unified Health System (SUS). Indeed, health regions constitute an essential organizational level for planning, coordination, and allocation of surveillance and care resources, including those related to TB control.

However, our unit of analysis was the municipality, which is nested within health regions. This choice allows for greater spatial granularity and precision in identifying local heterogeneities and persistent clusters that may otherwise be masked in broader regional analyses. Brazil currently comprises 450 health regions, whose boundaries often include municipalities with diverse epidemiological, demographic, and socioeconomic profiles. Aggregating data at that level could therefore introduce spatial smoothing effects and reduce the sensitivity to detect localized high-risk areas—one of the key aims of this study.

While we recognize that analyses at the health-region level could offer valuable insights for operational planning within the SUS framework, incorporating this additional layer would substantially expand the scope and complexity of the present work. Our focus was to provide municipal-level evidence that can, in turn, inform and be integrated into regional and state-level TB control strategies.

We appreciate the reviewer’s observation and agree that future studies could build upon our findings by explicitly aligning spatial analyses with health-region structures to further strengthen the connection between epidemiological evidence and the operational organization of the SUS.

- One point that we cannot avoid debating is the COVID-19 pandemic. The abrupt drop in the time series in 2020 and 2021 was mentioned by the authors in the paper and provides an interesting and factual view, since Brazil was one of the countries that recorded the most deaths from the disease, and, in places like the Amazon, there were total collapses of the health system. Another unexplored issue is the institutional dismantling of the SUS, which under Bolsonaro's government has seen major institutional attacks and changes of ministers and officials.

**Response:**

We appreciate the reviewer’s insightful comment highlighting the importance of contextualizing the pandemic’s impact within the broader political and institutional dynamics of Brazil’s SUS. We agree that both the abrupt decline in PTB notifications during 2020–2021 and the structural weakening of the health system observed in recent years are interrelated phenomena that have shaped the observed epidemiological patterns.

To address this valuable point, we expanded the Discussion to incorporate this context. We noted that after 2014–2015, a growing trend in PTB incidence in several regions coincided with the implementation of fiscal austerity measures that progressively undermined public-health financing and system responsiveness. This process of institutional erosion likely contributed to the slowdown and subsequent reversal of earlier TB control gains.

We also emphasized that the COVID-19 pandemic further intensified these vulnerabilities, exposing the fragility of Brazil’s health infrastructure and governance. The health-system collapse in parts of the Amazon region exemplified how resource shortages, decentralization challenges, and weakened federal coordination disrupted essential TB surveillance and treatment services. Moreover, the institutional dismantling of the SUS under the Bolsonaro administration, characterized by ministerial instability, budget restrictions, and the federal government’s denialist stance during the pandemic, exacerbated these effects, as evidenced by recent analyses demonstrating a measurable “Bolsonaro effect” (/doi.org/10.1371/journal.pone.0288894) on health outcomes and vaccination coverage

Accordingly, the following paragraph was inserted into the Discussion (lines 377–389):

“Complementarily, the increasing trends observed in certain states and regions after 2014 may be associated with fiscal austerity policies that gradually weakened public health financing and reduced the responsiveness of the SUS [41]. As noted, these vulnerabilities were further aggravated during the COVID-19 pandemic. Moreover, political instability and the dismantling of health governance structures in recent years—characterized by successive changes in the Ministry of Health leadership and the circulation of anti-scientific rhetoric—further undermined the capacity of the system to meet population needs. This context had a particularly strong impact in historically vulnerable regions such as the Amazon, where health-system collapse became an imminent risk. Recent evidence supports this interpretation, demonstrating that political denialism contributed to increased mortality, declining vaccination coverage, and the weakening of Brazil’s National Immunization Program [42], factors that can also directly affect TB control by undermining essential surveillance, prevention, and continuity-of-care efforts.

…”

This addition responds directly to the reviewer’s concern by integrating the effects of austerity, pandemic disruptions, and institutional fragility into the broader discussion of TB trends and control challenges in Brazil.

- As a final addendum, I think it's relevant that the research brought up the debate on the 2030 Agenda and the complicated situation that Brazil currently finds itself in. In the future, the authors could include a more in-depth analysis with typologies of the municipalities that have historically shown a drop in the PTB trend and which are, or are not, in line with the global agenda. A municipality that has shown a consecutive increase in recent years may be within the limit stipulated by the 2030 Agenda, sparking new debates on the policy.

**Response:**

We sincerely thank the reviewer for this thoughtful and valuable observation. We agree that aligning our findings with the United Nations 2030 Agenda and the Sustainable Development Goals (SDGs), particularly Goal 3, which aims to end the TB epidemic, represents an important direction for future analyses.

In future research, we intend to incorporate typologies of municipalities that have historically shown declining or rising PTB trends to assess their alignment with global TB elimination targets. We greatly appreciate this suggestion, which will be taken into consideration in the design of our forthcoming studies focusing on the intersection between spatial epidemiology, health governance, and sustainable development in Brazil.

Furthermore, we incorporated new information into the Discussion regarding a recent study published in The Lancet Global Health (2025; DOI: 10.1016/S2214-109X(25)00321-3), which can help advance the debate on the 2030 Agenda for TB elimination. This material was added to lines 361–368 to strengthen the evidence presented. The added passage reads:

“In the same direction, recent evidence shows that scaling up screening and preventive treatment among priority populations in Brazil could prevent approximately 15% (95% CI: 12.8–17.5%) of all TB episodes by 2050 and avert an estimated 1.8 million DALYs (95% CI: 1.2–2.3) over the period. Although expanding these interventions would require up to 62% of Brazil’s national TB budget in 2030, costs would gradually decline and remain well below cost-effectiveness thresholds. Notably, the study estimates that every dollar invested would return roughly 51 dollars to society, reinforcing that preventive interventions are highly cost-effective and yield substantial social, economic, and health benefits [35].”

This addition strengthens the discussion by providing updated evidence with direct implications for Brazil’s trajectory toward achieving the WHO End TB targets and the 2030 Agenda.

- Finally, I congratulate the authors on the work they have presented, with many figures, tables, and a wealth of supplementary material that fully supports the reading and understanding of the work. Studying Brazil in its plurality and regional diversity spread over 5570 municipalities is a major challenge. Combating tuberculosis and other diseases in Brazil requires coordinated action, and, with this in mind, this work offers relevant insights for the study of tuberculosis in Brazil.

Without further ado, I am available for future dialogues to discuss the relationship between health and geography for future work—I believe that this relationship is fundamental for strengthening the SUS.

**Response:**

We deeply appreciate the reviewer’s generous and encouraging comments. It is gratifying to know that the study’s comprehensiveness and analytical depth were well received. We also share the reviewer’s view that the intersection between health and geography is fundamental to understanding and strengthening the SUS. Spatial thinking provides essential insight into how structural and territorial factors shape access to care, exposure, and disease persistence.

We sincerely thank the reviewer for the offer of future dialogue and collaboration on this topic. Exploring the spatial and territorial dimensions of health—particularly within the SUS framework—remains a key priority in our ongoing and future research.
